# Supplementary material for: Efficient robot navigation inspired by honeybee learning flights
Source: Nature. 2026 May 13;653(8116):1039–46. doi: 10.1038/s41586-026-10461-3 (PMC13216067; doi:10.1038/s41586-026-10461-3)
Supplement: Supplementary file 1 — This file contains Supplementary Sections 1–14 including Supplementary Figs. 1–33 and additional references. [file 41586_2026_10461_MOESM1_ESM.pdf]

nature portfolio

https://doi.org/10.1038/s41586-026-10461-3

Supplementary information

Efficient robot navigation inspired by honeybee learning flights

In the format provided by the authors and unedited

Supplementary Information

EFFICIENT ROBOT NAVIGATION INSPIRED BY HONEYBEE LEARNING FLIGHTS

D. Ou[1], J.J. Hagenaars[1], M. Jankowski[1], M.V.M. Firlefyn[1],

C. De Wagter[1], F.T. Muijres[2], J. Degen[3], and G.C.H.E. de Croon[1]*

1. Micro Air Vehicle laboratory, Control and Operations, Faculty of Aerospace Engineering, Delft University of Technology, the Netherlands

2. Experimental Zoology Group, Wageningen University, the Netherlands.

3. Navigation Biology Group, Institute of Biology and Environmental Sciences, Carl von Ossietzky University of Oldenburg, Germany.

The navigation strategy proposed in the main article relies on a neural network to estimate the home location in a learned homing area. If the proposed strategy is to be deployed in real-world applications, trust is needed in the robustness of the neural network learning. Although neural networks are often considered "black boxes", analysis of their inner workings is possible and can bring insights into their capabilities and limitations. As a first step, in this supplementary information, we provide further analysis on the learning of home vectors within the learned homing area. The analysis is based both on theory and simulations, assuming a simplified model of the environment and the robot's perception. It treats both the information that can be exploited by the neural network and – to the extent possible – how this information is actually processed or represented in the network.

In section SI-1, we introduce the model and simulator used for the analysis., In this first section, we analyse a case in which the network has access to a global heading and can uniquely identify landmarks. Subsequently, in sections SI-2 to SI-4, we render the learning task increasingly difficult by reducing and deteriorating the information available to the network, studying the effects of this on the neural network homing strategy and performance. The analysis gives insight into why the neural network can generalize beyond the positions visited during the learning flight. Moreover, it shows that the use of path integration for determining the supervised learning targets leads to tortuous visual homing trajectories within the learned homing area. Next, in SI-5 we verify the results from the simplified, non-visual simulator by performing experiments in a visual simulator of an elementary environment with cylindrical landmarks.

Subsequently, we provide further supplementary information on the experiments reported on in the main article. In SI-6 we evaluate two different flight patterns that the real-world robot can take to sample the images during learning. Then, in SI-7, we re-analyse honeybee data in order to verify whether the flight trajectories of honeybees also have different tortuosity inside and outside of the learned homing area. In SI-8 we perform a preliminary investigation of how the used visual homing neural networks deal with opaque and dynamic obstacles in the learned homing area. This is followed in SI-9 by the application of a Simultaneous Localization And Mapping (SLAM) method to one of the experimental environments, in order to assess its memory requirements. Furthermore, in SI-10, more information is provided on the visually realistic simulator for the visual homing simulation experiments. In SI-11 we investigate the path integration accuracy of our robotic system, and other path integration methods. Moreover, in SI-12, we study a mushroom-body inspired visual homing method. In SI-13 we report on the comparison between the compact and attentional neural networks for visual homing. Finally, in SI-14 we study the visual homing performance of the neural network in additional simulation environments.

SI-1: Point-landmark-based model for learning homing vectors

In the main article, we introduced a self-supervised learning scheme of a neural network that transforms omnidirectional images to a vector pointing at the home location. Here, we investigate what such a network may be learning with the help of a simplified simulator. The main simplification is perceptual; We consider landmarks to be points, located in a 2D plane. This term implies that in our model, the robot will only see and hence exploit landmarks that are observed at a single location, like a tree. Moreover, whereas trees can be asymmetric in shape, in the simulator we will consider landmarks to have no perceivable orientation, like cylinders. These assumptions form a simplification of what a neural network can learn from images, since they exclude other information like landmark orientation, line features (such as furrows in a field), or features that are not spatially local but capture the gist of a scene.

In the model, we assume without loss of generality that the home location is at the origin of the axis system, i.e. at $(x_h, y_h) = (0,0)$. We assume that the home itself is not visible to the simulated robot. Furthermore, the environment contains $L \geq 1$ landmarks, with locations in the 2D plane. The robot can observe the distance and angle to all landmarks, in its own body reference frame. Moreover, initially we assume that the robot can uniquely identify each landmark. Visually, the angle to a landmark is available via its horizontal position in the image, the distance can be inferred from properties such as the object size or the vertical position in the image where the object touches the ground, and the identity of a landmark can be related to its unique shape, texture, or colour.

Our analysis starts with a robot that has access to a global heading. Two ways of obtaining the global heading are to observe the sun and correct for its position over time or to use a magnetic sense. This allows the robot to rotate the observations of landmarks into the world frame. Hence, in this initial model, the neural network will be provided with observations $(\Delta x_{w,l}, \Delta y_{w,l})$ for landmarks $l \in \{1, \ldots, L\}$:

$$\Delta x_{w,l} = x_l - x$$

$$\Delta y_{w,l} = y_l - y$$

, where the subscript w indicates the world frame and l stands for landmark. The coordinate (x, y) is the position of the robot. Moreover, we start with landmarks that are uniquely identifiable by the robot's visual system. If we think in terms of trees, then this means that every tree is unique to the robot due to its type, shape, etc. For the neural network, this means that it has $2L$ inputs. It also has two outputs that indicate a home vector $\boldsymbol{h}_r = (\Delta x_{w,h}, \Delta y_{w,h}) = (x_h - x, y_h - y) = (-x, -y)$.

Under the above assumptions, a single landmark suffices to determine the home location. Intuitively, the reason for this is that (1) the robot can observe the relative location to the landmark in the world frame, and (2) the landmark has a fixed position with respect to the home in the world frame. These vectors can be added to give the home location. The equation that captures this intuition is:

$$\boldsymbol{h}_r = (\boldsymbol{l} - \boldsymbol{p}) - (\boldsymbol{l} - \boldsymbol{h}) = (\boldsymbol{h} - \boldsymbol{p})$$

Where $\boldsymbol{l}$ is the landmark position, $\boldsymbol{p}$ the robot position, and $\boldsymbol{h}$ the home location, all in the world frame. Furthermore, $(\boldsymbol{l} - \boldsymbol{p})$ is the observation available to the robot, i.e., the coordinate of the landmark with respect to the robot. This formulation of the observation implies that the robot has access to a global heading, as it is expressed in terms of world frame coordinates without dependence on the robot's heading ψ. Furthermore, $(\boldsymbol{l} - \boldsymbol{h})$ is the coordinate of the landmark in the home frame. Since we assume $\boldsymbol{h} = (0,0)$, we get:

$$\boldsymbol{h}_r = -\boldsymbol{p}$$

Which is the negative robot coordinate in the world frame.

We implemented the model in Python and trained a neural network to map observations $\boldsymbol{o} = (\boldsymbol{l} - \boldsymbol{p})$ to target directions $\boldsymbol{h}_r$. Specifically, we took 1000 uniformly random samples in the area $x, y \in [-10,10]$. We trained a perceptron with bias to map the observation of a single landmark to the home location. A perceptron is a feedforward neural network without hidden layer. The network has two input neurons for the landmark observation and one bias input of value 1. Since it has two output neurons, the network only has six connections with corresponding weights (three inputs times two outputs). For training, we used the Mean Squared Error (MSE) loss, and the Adam optimizer, with a learning rate of 0.05. We used a single landmark at the location $\boldsymbol{l} = (3,3)$. The training loss was close to zero, $1.33\ 10^{-12}$.

In the figure below, we show the predictions of the perceptron at a grid in the environment. As expected, we get a radial vector field with all vectors pointing at the home location.

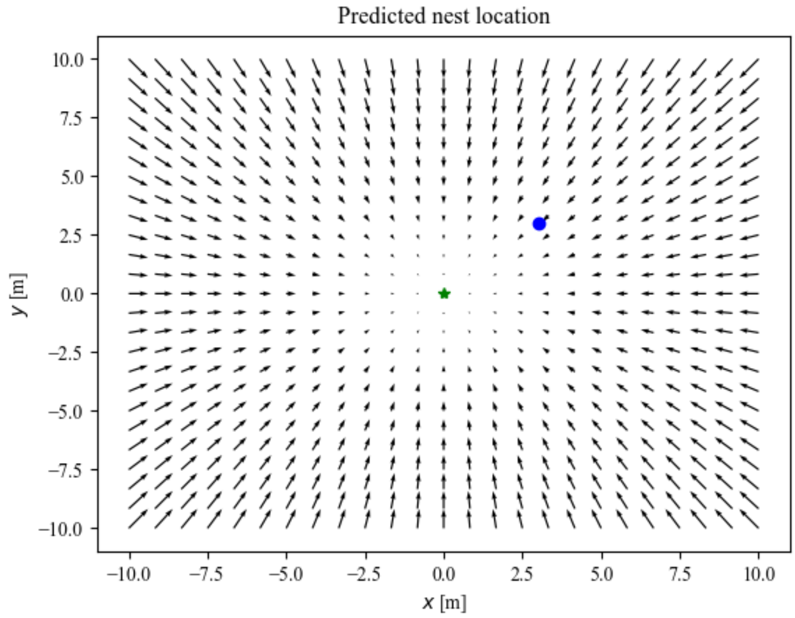

Figure SI-1: Predicted homing directions of a trained perceptron that has access to a global heading. One landmark suffices for determining the home location. Please note that the arrows are scaled-down versions of the actual predictions for a neater visualization. The actual vectors generally point directly to the origin in this case.

We also analysed the four input weights and the two bias weights. Rounded to two decimals, the input weights are:

$$\begin{bmatrix} w_{1,1} & w_{1,2} \\ w_{2,1} & w_{2,2} \end{bmatrix} = \begin{bmatrix} 1.00 & 0 \\ 0 & 1.00 \end{bmatrix}$$

And the bias weights are: $[b_1 \quad b_2] = [-3.00 \quad -3.00]$. The weights show that the perceptron exactly implements the expected formula, first displacing to the landmark with the weights from the observation and then subtracting the position of the landmark via the bias weights.

This leads to the following important insight into the network's generalization capability. With these weights, the neural network will perfectly generalize to unseen positions within and outside of the learned area. This insight has a relation to the real world, as the underlying assumptions of this model are not implausible. The most likely problem in the real world is that at larger distances, the landmark will no longer be visible, due to it being occluded or too small in view. Moreover, identifying a landmark and estimating the distance to it, will become increasingly difficult at larger distances.

SI-2: Learning to home without a global heading

In the previous section, the task of the neural network was substantially simplified by the landmark observation being relative to the robot, but in a global orientation frame of reference. In this section, we study the case in which the robot does not have access to a global heading estimate. This may happen when, e.g. the sky is almost completely covered with clouds.

Concretely, in the simulator, we now generate observations for training and testing by giving the robot a random orientation ψ in the interval $[0,2\pi]$. The neural network now receives for each landmark a two-dimensional observation $\boldsymbol{o}$:

$$\boldsymbol{o} = \boldsymbol{R}(\boldsymbol{l} - \boldsymbol{p})$$

$$\boldsymbol{R} = \begin{bmatrix} \cos(\psi) & -\sin(\psi) \\ \sin(\psi) & \cos(\psi) \end{bmatrix}$$

, where there now is a dependence of the observation on the clockwise positive heading ψ.

This case is extra relevant, since it is closest to the real robot experiments, in which we do not use any global orientation information external to the image. This means that we do not, e.g., use a magnetometer to rotate the omnidirectional images to always align the centre pixel coordinate with the North direction. Please note that it is still possible in visual experiments for a neural network to extract a global orientation from distant landmarks such as a large mountain. Although the case does not exploit all information that is typically present for honeybees, learning to home without using a global heading reference does bring extra robustness. Hence, it may be biologically relevant as well, with honeybees potentially ignoring global heading information for more robust view memory. For this setting, it becomes important how many landmarks there are. We first analyse the case of one landmark and then of two or more landmarks.

One landmark

If there is only one point landmark, the robot has no means of knowing on which side of the landmark it is. This means that if the robot tries to determine its position in the world frame, it actually only knows at which distance from the landmark it is.

In this case, we see a clear difference between a perceptron and a neural network with hidden layers. The perceptron can only make linear transformations of the input, and will always point to the landmark location, no matter where it is located. A neural network with a hidden layer can learn non-linear transformations and is able to tune the response to the landmark location, choosing a specific distance to the landmark.

Below, we show results for a multi-layer perceptron. The input layer has two input neurons to represent the observation to the single landmark, and an input bias with value 1. The network has a single hidden layer of 100 neurons, all with a ReLU activation function. Also this layer has a bias. The output layer with two output neurons has a linear activation function. The two outputs represent the home vector in the robot's frame of reference.

The training error depends on the location of the landmark. When the landmark is located at $(3,3)$, the error is 6.77. The top row in Figure SI-2 shows the directions of the neural network predictions on a grid, for different headings. For this landmark close to the home location, the neural network learns to point in the direction of the landmark. The bottom row in Figure SI-2 shows the results when the landmark is located at $(10,10)$. The final training error is 16.23. In that case, during training the robot was always to the bottom left of the landmark in the world frame. If a robot were to follow the neural network vectors, it would end up at a certain distance from the landmark. Indeed, one can see a quarter of a circle around the landmark where the magnitude of the predicted homing vector is close to zero. It

is intriguing that the network does not choose a distance that ever brings the robot exactly to the nest. Below, we will explain this by means of a mathematical analysis.

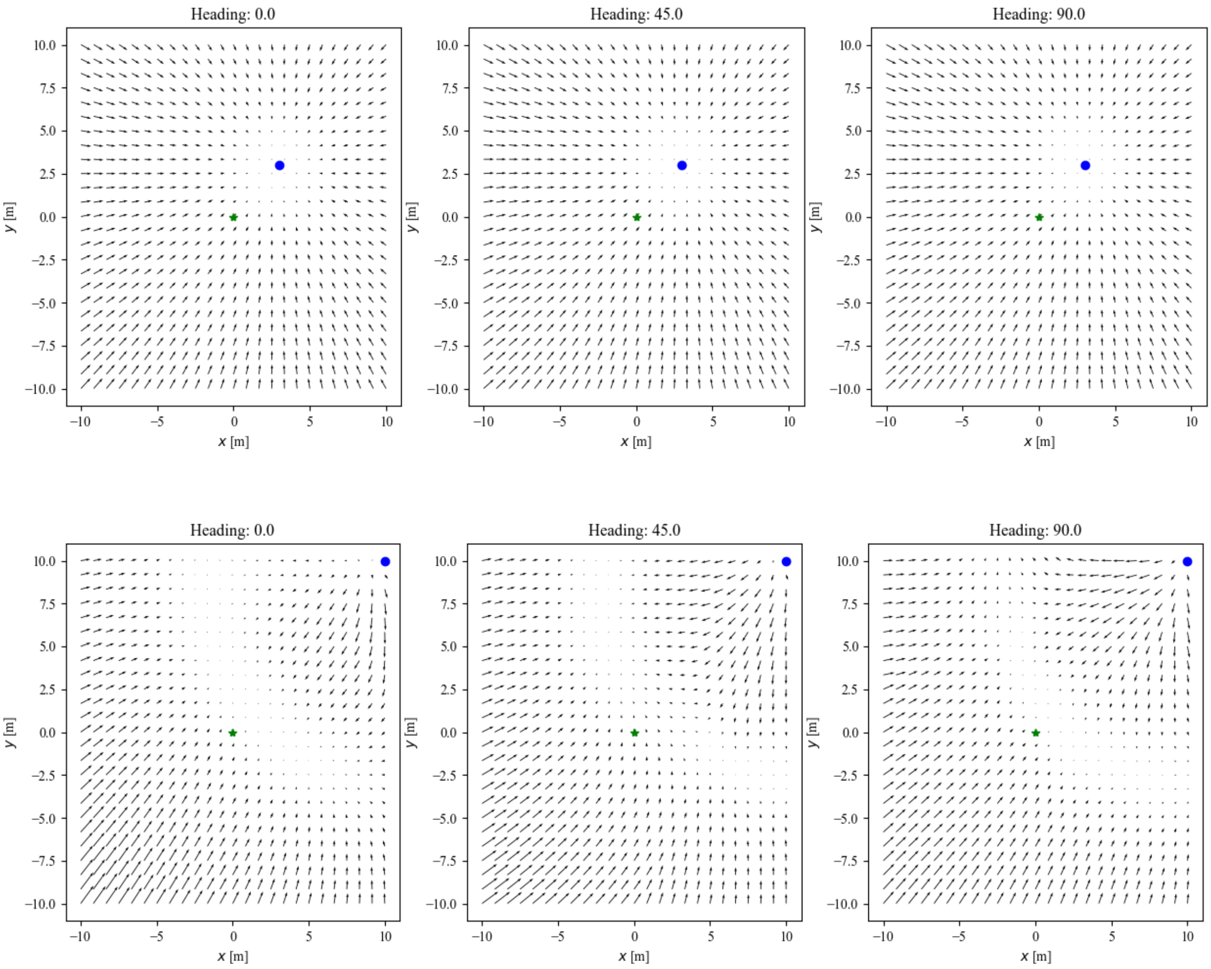

Figure SI-2: Neural network predictions for different headings when the robot has no access to a global heading and there is only one landmark. *Top row:* Predictions when the landmark is located at (3,3). The home location is indicated by the green star and the landmark by the blue circle. *Bottom row:* Predictions when the landmark is located at the edge of the learning area, at (10,10).

Since the robot does not have access to its global heading and cannot use the single landmark to infer this heading, it can only choose to go to a given distance of the landmark. What distance is optimal, depends on the region from which the training samples are taken. If the robot moves in an infinitely large space and the landmark has a finite distance to the nest, then it is always best to move towards the landmark. However, if the landmark approaches the limits of a finite region in which the samples are taken, then it becomes better to keep a certain distance from the landmark.

We can derive mathematically what this optimal distance is, with the help of a line integral. Specifically, we are looking for the radius r that will minimize the expected value of our mean squared error loss function:

$$\mathbb{E}[\mathcal{L}(\hat{x},\hat{y})] = \mathbb{E}[(\hat{x} - h_{x,b})^2 + (\hat{y} - h_{y,b})^2]$$

, where $\hat{x}$ is the estimated and $h_{x,b}$ is the true relative x-coordinate of the home location in the robot's body frame. As an example, we will now analyse the case of the landmark at the full top right of the environment, i.e., at (10,10). In this case, if the robot moves to a given distance to the landmark, it

will end up at the bottom left quarter of the associated circle around the landmark. It cannot be predicted beforehand at which location on this quarter circle the robot will end up. Hence, in order to obtain the expected loss, we need to integrate the error along all the positions of the quarter circle and divide it by the length of the quarter circle, i.e. $\frac{1}{2}\pi r$, where r is the radius of the circle. We do this by means of a line integral, where for mathematical convenience, we will place the landmark at coordinate $(0,0)$ and the home at location $(h_x, h_y) = (-10, -10)$. Hence, the parametric equation for a circle around the landmark, with parameter t, becomes:

$$\boldsymbol{r}(t) = (r\,cos(t), r\,sin(t))$$

$$f(\boldsymbol{r}(t)) = (r\,cos(t) - h_x)^2 + (r\,sin(t) - h_y)^2$$

, where $f(\boldsymbol{r}(t))$ is the loss at the location $(x, y) = (r\,cos(t), r\,sin(t))$. Integrating it along the quarter circle gives:

$$\mathbb{E}_r[\mathcal{L}(\hat{\boldsymbol{x}}, \hat{y})] = \int_{t=\pi}^{\frac{3}{2}\pi} f(\boldsymbol{r}(t))\|\boldsymbol{r}'(t)\|\,dt \,/\, \frac{1}{2}\pi r =$$

$$\int_{t=\pi}^{\frac{3}{2}\pi} \left((r\,cos(t) - h_x)^2 + (r\,sin(t) - h_y)^2\right)\|\boldsymbol{r}'(t)\|\,dt \,/\, \frac{1}{2}\pi r$$

, and since $\|\boldsymbol{r}'(t)\| = \sqrt{r^2cos(t)^2 + r^2sin(t)^2} = \sqrt{r^2(cos(t)^2 + sin(t)^2)} = \sqrt{r^2} = r$, we have:

$$\int_{t=\pi}^{\frac{3}{2}\pi} \left((r\,cos(t) - h_x)^2 + (r\,sin(t) - h_y)^2\right)r\,dt \,/\, \frac{1}{2}\pi r =$$

$$\int_{t=\pi}^{\frac{3}{2}\pi} \left((r\,cos(t) - h_x)^2 + (r\,sin(t) - h_y)^2\right)dt \,/\, \frac{1}{2}\pi =$$

$$\int_{t=\pi}^{\frac{3}{2}\pi} \left(r^2\,cos(t)^2 - 2\,r\,h_x\,cos(t) + {h_x}^2 + r^2\,sin(t)^2 - 2\,r\,h_y\,sin(t) + {h_y}^2\right)dt \,/\, \frac{1}{2}\pi =$$

$$\int_{t=\pi}^{\frac{3}{2}\pi} \left(r^2 + {h_x}^2 + {h_y}^2 - 2\,r\,h_x\,cos(t) - 2\,r\,h_y\,sin(t)\right)dt \,/\, \frac{1}{2}\pi$$

, because $r^2cos(t)^2 + r^2sin(t)^2 = r^2$. This leads to the integral:

$$g(r) = \mathbb{E}_r[\mathcal{L}(\hat{x}, \hat{y})] = \left[\left(r^2 + {h_x}^2 + {h_y}^2\right)t - 2\,r\,h_x\,sin(t) + 2\,r\,h_y\,cos(t)\right]_{\pi}^{\frac{3}{2}\pi} / \frac{1}{2}\pi =$$

$$\left(\frac{1}{2}\pi\left(r^2 + {h_x}^2 + {h_y}^2\right) + 2\,r\,h_x + 2\,r\,h_y\right) / \frac{1}{2}\pi =$$

$$r^2 + {h_x}^2 + {h_y}^2 + \frac{4}{\pi}r\,(h_x + h_y)$$

If we want to minimize the expected loss, $g(r)$, we can set its derivative with respect to r to zero:

$$g'(r) = 0$$

$$g'(r) = 2r + \frac{4}{\pi}(h_x + h_y) = 0$$

$$r^* = -\frac{2}{\pi}(h_x + h_y) = \frac{40}{\pi}$$

, where the last equation comes from the fact that when the landmark is at $(0,0)$, the nest is at a negative location: $(h_x, h_y) = (-10, -10)$. However, up to that equation, the analysis is valid for any case in which the landmark is at the top right of an environment, so that all chosen radii form quarter circles.

In the figure below, we show the outputs of the network (black arrows), the optimal radius r^*(black dashed circle, green loss $\mathcal{L}^*$), and the mean loss for circles of different radii from lower errors (green) to higher errors (red). The theoretically optimal radius r^* has a lower error $\mathcal{L}$ than the other radii. Moreover, the neural network, which was trained to minimize the mean squared error $\mathbb{E}[\mathcal{L}(\hat{x}, \hat{y})]$, points to the circle of this radius.

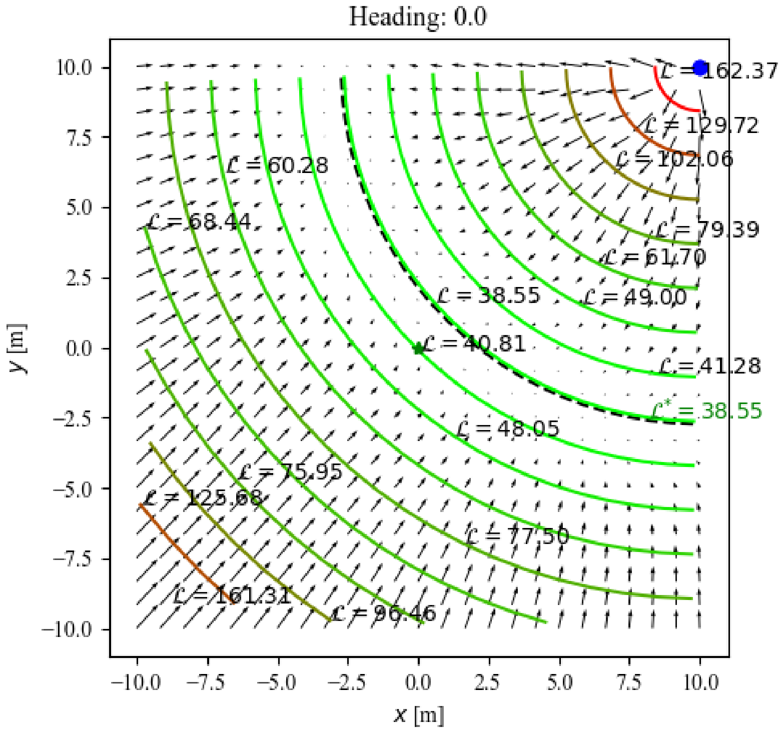

Figure SI-3: Output vectors from the trained neural network, together with the optimal radius from the landmark (black dashed line) and numerically determined loss values of the valid parts of the circle for other radii. The network neatly points to the theoretical optimum.

Interestingly, if we allow the robot to be located in any direction from the landmark, we get the following result (note that we have the same formula for $g(r)$, but change the integration limits to $0{,}2\pi$):

$$g(r) = \mathbb{E}_r[\mathcal{L}(\hat{x}, \hat{y})] = \left[\left(r^2 + {h_x}^2 + {h_y}^2\right)t - 2\,r\,h_x\,sin(t) + 2\,r\,h_y\,cos(t)\,\right]_0^{2\pi} / \frac{1}{2}\pi =$$

$$\left(2\pi\left(r^2 + {h_x}^2 + {h_y}^2\right)\right) / \frac{1}{2}\pi =$$

$$4\,(r^2 + {h_x}^2 + {h_y}^2)$$

If we want to minimize the expected loss, $g(r)$, we can set its derivative to zero:

$$g'(r) = 0$$

$$g'(r) = 8r = 0$$

$$r^* = 0$$

This shows that when the robot can be located in an infinitely large space around the nest, it is best to go straight to the landmark. This corresponds to the strategy followed by the neural network when the landmark is located closer to the nest. Then the space of the entire region from which the neural network receives samples is relatively large compared to the distance between the landmark and the nest.

Intuitively, the strategy of moving towards a landmark will generalize outside of the learning region. Here, we switch back to a trained perceptron and investigate its weights to verify whether the neural network weights support this. When the landmark is located at (3,3), the perceptron weights are as follows:

$$\begin{bmatrix} w_{1,1} & w_{1,2} \\ w_{2,1} & w_{2,2} \end{bmatrix} = \begin{bmatrix} 0.79 & 0 \\ 0 & 0.79 \end{bmatrix}$$

And the bias weights are: $[b_1 \quad b_2] = [-0.02 \quad -0.05]$. Between different learning runs, the bias weights vary a bit, but they are always close to zero. The result of these weights is that the robot will move towards the landmark, something that will generalize outside of the learning region. However, we were initially puzzled by the fact that the weights are lower than one, i.e., they do not map directly to the landmark location. The reason for this turns out to be the shape of the training area, which is square. Since there are more points to the bottom left of the landmark, it pays off in terms of the MSE to go only part of the way to the landmark position. With weights of 0.79 more points will end up closer to the home location. This can be seen in the left plot in Figure SI-4, which shows the end points of the neural network predictions, when the network is evaluated at a grid spanning the entire region of $x, y \in [-10,10]$.

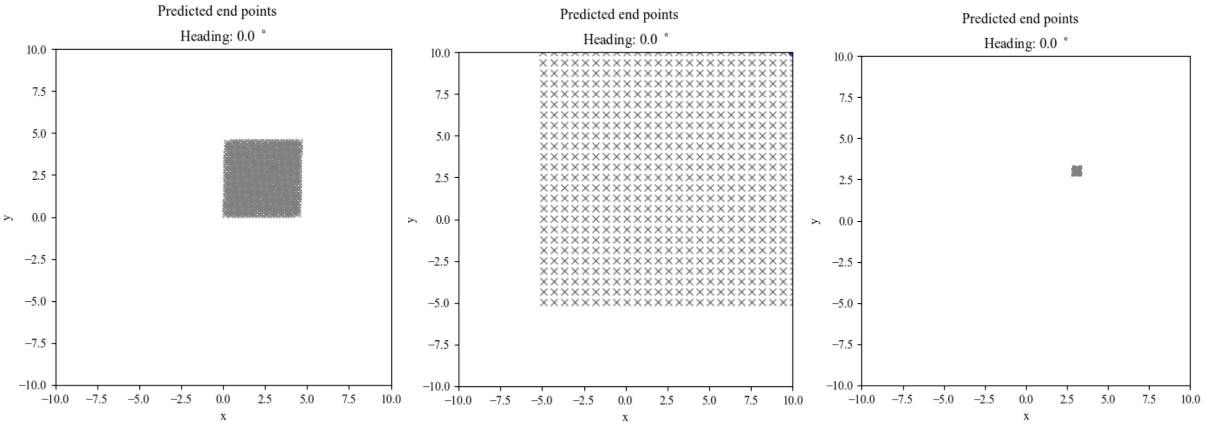

Figure SI-4: End points of the neural network predictions for different landmark positions and different training areas. *Left:* With the landmark at (3,3) and the training region $x, y \in [-10,10]$, the network linearly scales the inputs so that a larger part of the end points is close to the home location. *Middle:* With the landmark at (10,10), the network scales the inputs less, in order to have end points close to the home location. *Right:* With the landmark at (3,3) and a circular learning region around the landmark, the network maps directly to the landmark.

The weights are even smaller when the landmark is at (10,10):

$$\begin{bmatrix} w_{1,1} & w_{1,2} \\ w_{2,1} & w_{2,2} \end{bmatrix} = \begin{bmatrix} 0.25 & 0 \\ 0 & 0.25 \end{bmatrix}$$

And the bias weights are: $[b_1 \quad b_2] = [0.04 \quad 0.03]$. The middle plot in Figure SI-4 shows the effect of this landmark location on the end points. Finally, if we use a circular training region that is centred at

the landmark, there is no sampling bias in the training set with respect to the home location. The perceptron weights are as expected, namely close to 1:

$$\begin{bmatrix} w_{1,1} & w_{1,2} \\ w_{2,1} & w_{2,2} \end{bmatrix} = \begin{bmatrix} 1.01 & 0 \\ 0 & 1.01 \end{bmatrix}$$

And the bias weights are: $[b_1 \quad b_2] = [0.05 \quad 0.01]$. This puts the prediction end points all very close to the landmark (Figure SI-4, right).

Two or more landmarks

When there are two or more landmarks, neural networks are able to map the observations directly to the home location again. This results in a radial flow field towards the home location. Intuitively, having two uniquely identifiable landmarks restores global heading information. The vector from one landmark to the other forms a type of "compass needle" that the network can exploit to find the home location.

Below, we show the vector field for a perceptron. It receives the observations for two landmarks, one at $(1,3)$ and the other at $(-2,5)$, and has an input bias. The perceptron is able to pinpoint the home location exactly.

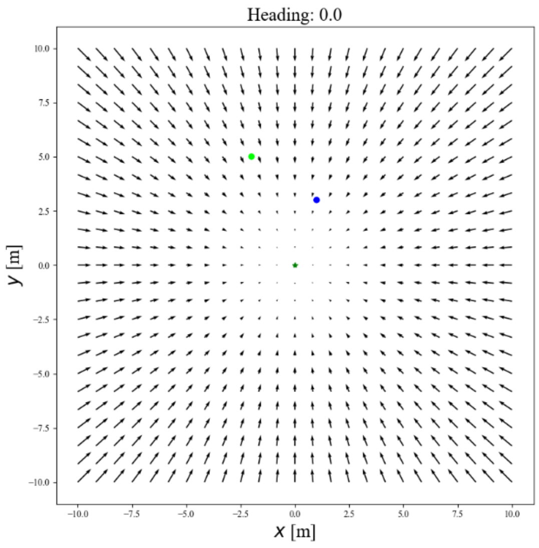

Figure SI-5: Perceptron homing predictions for the case of two unique landmarks.

In order to see how generalizable the solution represented by the neural network is, we analyse the perceptron weights, which are:

$$\begin{bmatrix} w_{1,1} & w_{1,2} & w_{1,3} & w_{1,4} \\ w_{2,1} & w_{2,2} & w_{2,3} & w_{2,4} \end{bmatrix} = \begin{bmatrix} 1.23 & 0.85 & -0.23 & -0.85 \\ -0.85 & 1.23 & 0.85 & -0.23 \end{bmatrix}$$

And the bias weights are: $[b_1 \quad b_2] = [0.00 \quad 0.00]$. Please remark that we have four inputs now, $(\Delta x_1, \Delta y_1, \Delta x_2, \Delta y_2)$, representing the relative locations with respect to the two landmarks in the robot's body frame. There are still two outputs for the homing vector, also represented in the robot body frame. Remarkably, when looking at the trained perceptron, the bias weights are zero.

It turns out that we can accurately predict the perceptron weights, given the landmark locations. Intuitively, the network represents a strategy in which it first moves to the centroid landmark location. Then, it uses the vector between the landmarks to get to the home location. To achieve this, it follows an appropriately rotated version of this vector.

Mathematically, this strategy can be expressed as follows. First, we determine the weights again to end up at the centre of the landmarks:

$$\boldsymbol{w}_1 = [[0.5, 0, 0.5, 0], [0, 0.5, 0, 0.5]]$$

Then, the network uses the vector between the landmarks, $\Delta\boldsymbol{l} = \boldsymbol{l}_1 - \boldsymbol{l}_2$:

$$\boldsymbol{w}_2 = [[1, 0, -1, 0], [0, 1, 0, -1]]$$

However, this vector may not be pointing at the nest location. So, we need to rotate this vector to point at the nest. The vector from the landmark centroid $\boldsymbol{c}$ to the nest is the constant vector:

$$\boldsymbol{c} = -\frac{\boldsymbol{l}_1 + \boldsymbol{l}_2}{2}$$

Using the cosine rule, we can find the fixed angle between $\boldsymbol{c}$ and $\Delta\boldsymbol{l}$:

$$\beta = acos(\frac{\boldsymbol{c}\,\Delta\boldsymbol{l}}{||\boldsymbol{c}||\,||\Delta\boldsymbol{l}||})$$

, which allows us to make the 2D rotation matrix $\boldsymbol{R}$:

$$\boldsymbol{R} = \begin{bmatrix} cos(-\beta) & -sin(-\beta) \\ sin(-\beta) & cos(-\beta) \end{bmatrix}$$

Rotating $\Delta\boldsymbol{l}$ is not enough, as the vector also needs to be scaled, with a factor:

$$a = \frac{||\boldsymbol{c}||}{||\Delta\boldsymbol{l}||}$$

We then take the dot product of the two blocks of $\boldsymbol{w}_2$ with $a\boldsymbol{R}$:

$$\boldsymbol{w}_3 = \begin{bmatrix} a\boldsymbol{R}\begin{bmatrix} 1 & 0 \\ 0 & 1 \end{bmatrix} & a\boldsymbol{R}\begin{bmatrix} -1 & 0 \\ 0 & -1 \end{bmatrix} \end{bmatrix}$$

, which finally leads to the predicted weights:

$$\boldsymbol{w}' = \boldsymbol{w}_1 + \boldsymbol{w}_3$$

Indeed, printing the predicted weights for the above landmark configuration gives:

Predicted weights:

$$\begin{bmatrix} w_{1,1} & w_{1,2} & w_{1,3} & w_{1,4} \\ w_{2,1} & w_{2,2} & w_{2,3} & w_{2,4} \end{bmatrix} = \begin{bmatrix} 1.23 & 0.85 & -0.23 & -0.85 \\ -0.85 & 1.23 & 0.85 & -0.23 \end{bmatrix}$$

This solution, which points directly at the home location, will generalize to other areas outside of the training area.

Hence, summarizing, if the robot has no access to any global heading information, two uniquely identifiable landmarks suffice for accurately pinpointing the home location.

SI-3: Homing with similar-appearance landmarks

In this section, we study the case in which landmarks are no longer uniquely identifiable. An example of this would be that all trees around the home would look the same to the robot, something which could potentially be the case with the very coarse insect vision. In this case, two landmarks no longer suffice for retrieving a global heading, since it is no longer clear in which direction the "compass needle" is pointing.

In our simulator, we achieve this by randomly shuffling the landmark observations each time a sample is presented to the network. This means that for instance the first two neurons sometimes represent the relative location of landmark one and sometime the relative distance to landmark two or three. Hence, the network cannot rely on the same landmark always being represented with the same inputs.

In this case, perceptron neural networks are only able to move to the centre of the presented landmarks. When gathering training samples in a circular region around the landmark centroid, the learned weights simply represent the average of the relative landmark vectors in the body frame.

Hence, we focus here on multi-layer neural networks. If we train a multi-layer neural network to estimate the home vector in the presence of two landmarks, it can only move to their centre location (Figure SI-6, top left). However, given three landmarks in a sufficiently propitious configuration, a multi-layer neural network can pinpoint the home location. A configuration that does not lend itself to this is the equilateral triangle. In this configuration, the neural network cannot distinguish between the different landmarks and can hence only move to the centre of the landmarks (Figure SI-6, top right). However, as soon as at least one landmark stands apart from the others, the neural network is able to estimate the home location. This is shown by the results when the landmarks form an isosceles triangle (bottom left) or a scalene triangle (bottom right).

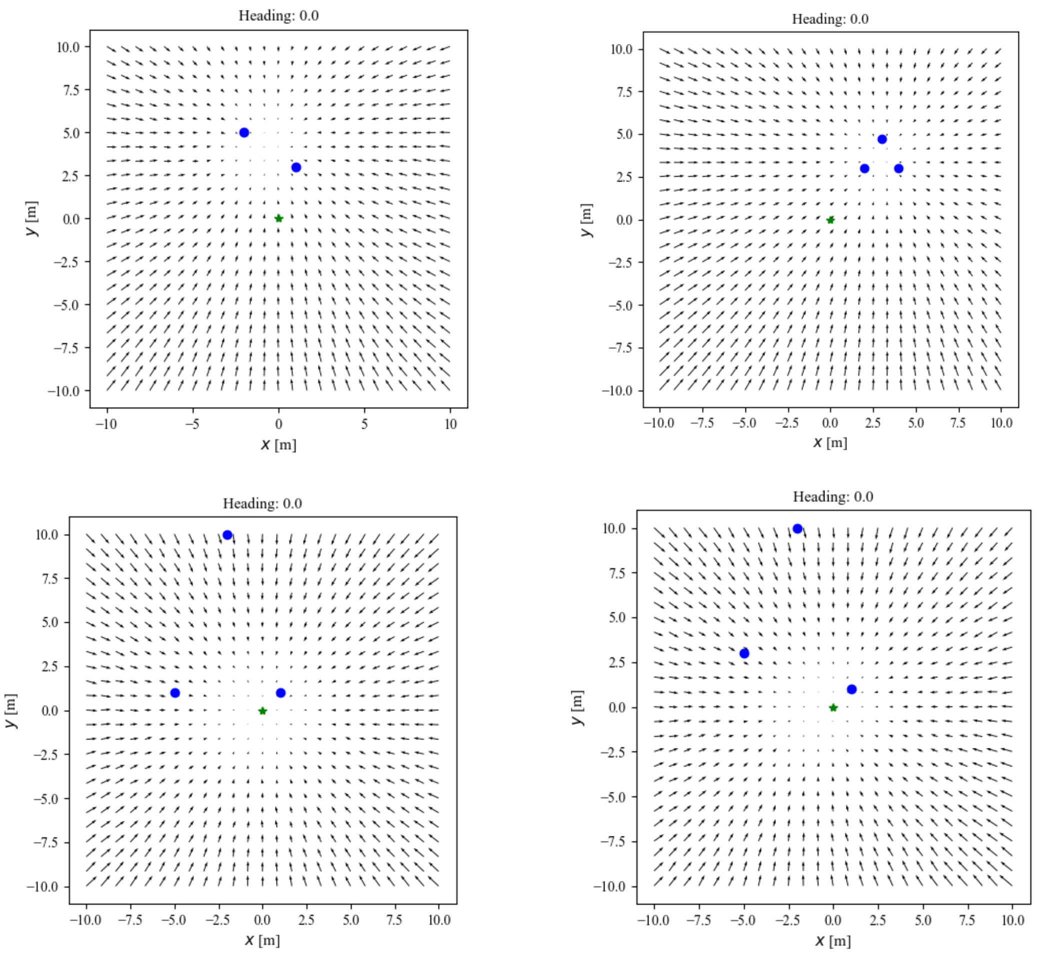

Figure SI-6: Predicted home vector directions for the case in which landmarks cannot be discerned from each other. *Top left:* Two landmarks. *Top right:* The landmarks form an equilateral triangle. *Bottom left:* The landmarks form an isosceles triangle. *Bottom right:* The landmarks form a scalene triangle.

Also in this case, we study what the neural network has learned. Ideally, one would like the hidden layer to be as small as possible for that, since it simplifies the analysis. However, the performance of the

network decreased substantially for lower numbers of hidden neurons. Hence, we will analyse the multi-layer perceptron with 100 hidden neurons.

First, we studied the activations of the hidden neurons, for different shuffling orders, orientations, and positions. Figure SI-7 shows the activations of the first 5 hidden neurons of a typical network trained on the isosceles triangle shown in the bottom left of Figure SI-6. Generally, there seem to be two types of neurons: “distance” neurons and “heading” neurons. The distance neurons have a graded response to the landmarks, which helps to scale the home vector. The response rotates with the heading, which means that the response can be used to move in a desired body direction towards the landmarks. However, the activation does not carry information on the global heading. Moreover, due to the ReLU activation function, the neurons activate at one side of the landmark configuration, providing directionality as to where to move. In contrast, the heading neurons only become active at a given global heading. They also mostly activate only for one shuffling order. Global heading information is exactly what is necessary to displace the estimated homing vector from the landmark centroid to the home location. Hence, we further investigated these neurons to see how they function.

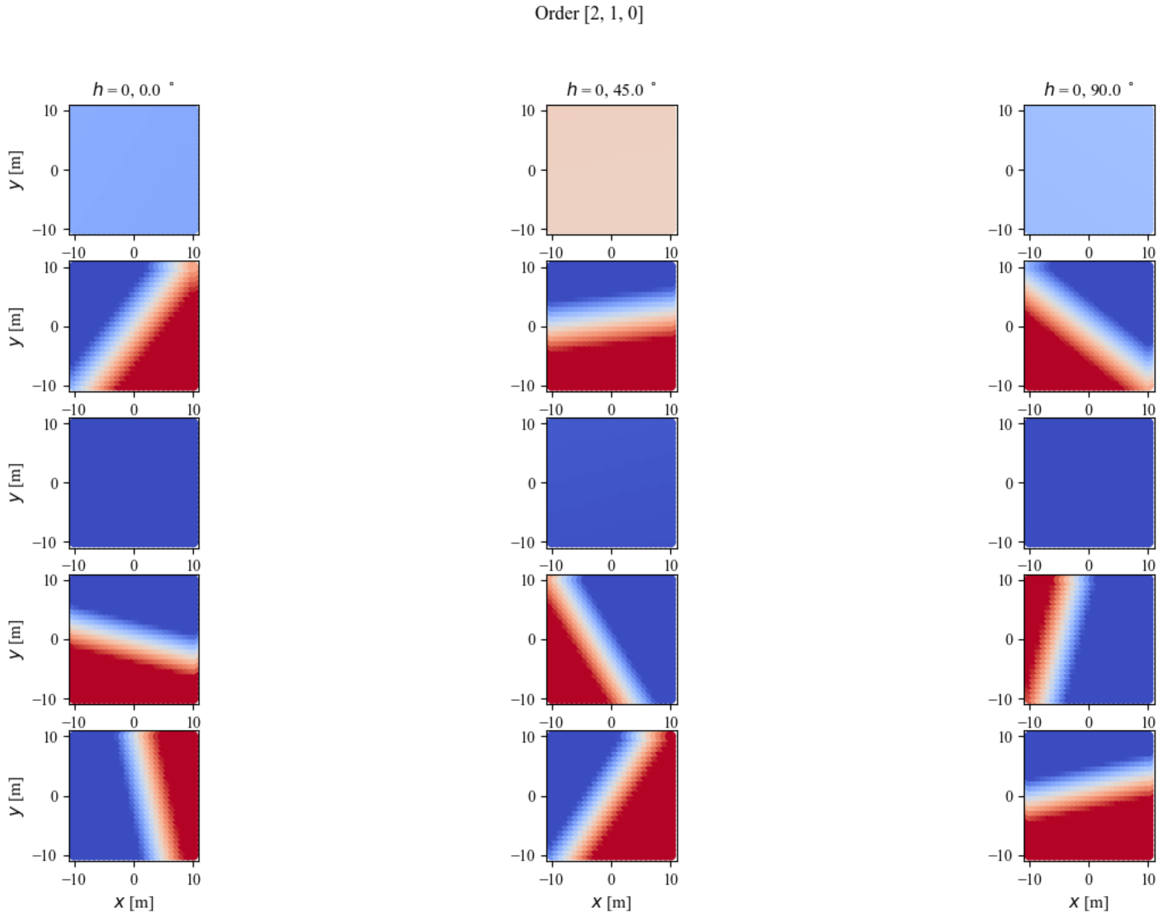

Figure SI-7: Activations over a grid of positions in the area $x, y \in [-10,10]$ of the first five hidden neurons of a neural network trained on the isosceles triangle of landmarks, for the shuffling order $[2,1,0]$ and for heading angles $\{0°, 45°, 90°\}$.

We first inspected the weights from the input layer to the hidden layer. Since the bias weights were really close to zero for these neurons, we will ignore them in the further analysis. Each hidden neuron has a connection to all six input neurons that represent the landmark observations. Strikingly, the weights from the input neurons to the heading neurons add up to zero for each coordinate. We will see how this eliminates the influence of the robot’s position, in the following mathematical analysis. We start with the observation formula for a single landmark i:

$$\begin{bmatrix} o_x^i \\ o_y^i \end{bmatrix} = \begin{bmatrix} \cos(\psi) & -\sin(\psi) \\ \sin(\psi) & \cos(\psi) \end{bmatrix} \begin{bmatrix} l_x^i - p_x \\ l_y^i - p_y \end{bmatrix}$$

The input to the hidden neuron, before ReLU is $h = h_x + h_y$. The x-part of the input is: $h_x = w_a o_x^1 + w_b o_x^2 + w_c o_x^3$, where 1, 2, and 3 refer to landmarks after they have been shuffled, so not a fixed order of landmarks. What we observe in heading neurons is that: $w_a = -(w_b + w_c)$, where the order of weights a, b, c varies over the different hidden neurons.

The consequence of this is very interesting, and can be seen when writing out h_x:

$$h_x = w_a \cos(\psi)\, l_x^1 - w_a \cos(\psi)\, p_x - w_a \sin(\psi)\, l_y^1 + w_a \sin(\psi)\, p_y$$

$$+ w_b \cos(\psi)\, l_x^2 - w_b \cos(\psi)\, p_x - w_b \sin(\psi)\, l_y^2 + w_b \sin(\psi)\, p_y$$

$$+ w_c \cos(\psi)\, l_x^3 - w_c \cos(\psi)\, p_x - w_c \sin(\psi)\, l_y^3 + w_c \sin(\psi)\, p_y$$

We can see that having $w_a = -(w_b + w_c)$ implies that h_x does *not* depend on position, since:

$$-w_a \cos(\psi)\, p_x - (w_b + w_c) \cos(\psi)\, p_x = -w_a \cos(\psi)\, p_x + w_a \cos(\psi)\, p_x = 0$$

And:

$$w_a \sin(\psi)\, p_y + (w_b + w_c) \sin(\psi)\, p_y = w_a \sin(\psi)\, p_y - w_a \sin(\psi)\, p_y = 0$$

So we end up with:

$$h_x = \cos(\psi)\, \overrightarrow{\boldsymbol{w}_x^T}\, \overrightarrow{\boldsymbol{l}_x} - \sin(\psi) \overrightarrow{\boldsymbol{w}_x^T}\, \overrightarrow{\boldsymbol{l}_y}$$

Likewise:

$$h_y = \sin(\psi)\, \overrightarrow{\boldsymbol{w}_y^T}\, \overrightarrow{\boldsymbol{l}_x} + \cos(\psi) \overrightarrow{\boldsymbol{w}_y^T}\, \overrightarrow{\boldsymbol{l}_y}$$

So that:

$$\begin{bmatrix} h_x \\ h_y \end{bmatrix} = \begin{bmatrix} \cos(\psi) & -\sin(\psi) \\ \sin(\psi) & \cos(\psi) \end{bmatrix} \begin{bmatrix} \overrightarrow{\boldsymbol{w}_x^T}\, \overrightarrow{\boldsymbol{l}_x} \\ \overrightarrow{\boldsymbol{w}_y^T}\, \overrightarrow{\boldsymbol{l}_y} \end{bmatrix}$$

This means that heading neuron inputs *do not* depend on the robot position $\boldsymbol{p}$. They do depend on heading and the specification of the weights within the constraint that there is a weight that is the negative of the sum of the other weights, $w_a = -(w_b + w_c)$.

The last equation contains a rotation matrix and the dot product between weights and the landmark observations. This leads to the insight that the neuron will respond maximally if the weights, rotated by the heading, maximally align with the landmarks. Plotting the weights to the heading neurons as arrows in weight space indeed reveals that the weight vectors approximately represent the isosceles triangle of the landmarks (Figure SI-8). Hence, the neural network weights here directly represent the geometric configuration of the landmarks.

Since the heading neurons only respond in a limited range around a given heading, the network needs a sufficient number of such neurons in order to estimate the home vector from any position and heading. Hence, we would expect the orientation of the largest weight vector to be uniformly distributed over all angles. Likewise, as the landmarks can be shuffled in any order, one would expect a uniform distribution of the largest vector over the different landmark positions in the input vector (i.e., over position 1, 2, and 3). Figure SI-9 shows that there is indeed a good spread over the various angles and landmark indices.

Furthermore, we also investigated the weights to the distance neurons. Here, we hypothesized that the weights would point roughly in the same direction, with a maximal response if the landmarks are located in that direction. Given the ReLU activation function, with only positive outputs, the weights to the output layer should point in roughly the same direction. This is indeed the case for most distance neurons (Figure SI-10).

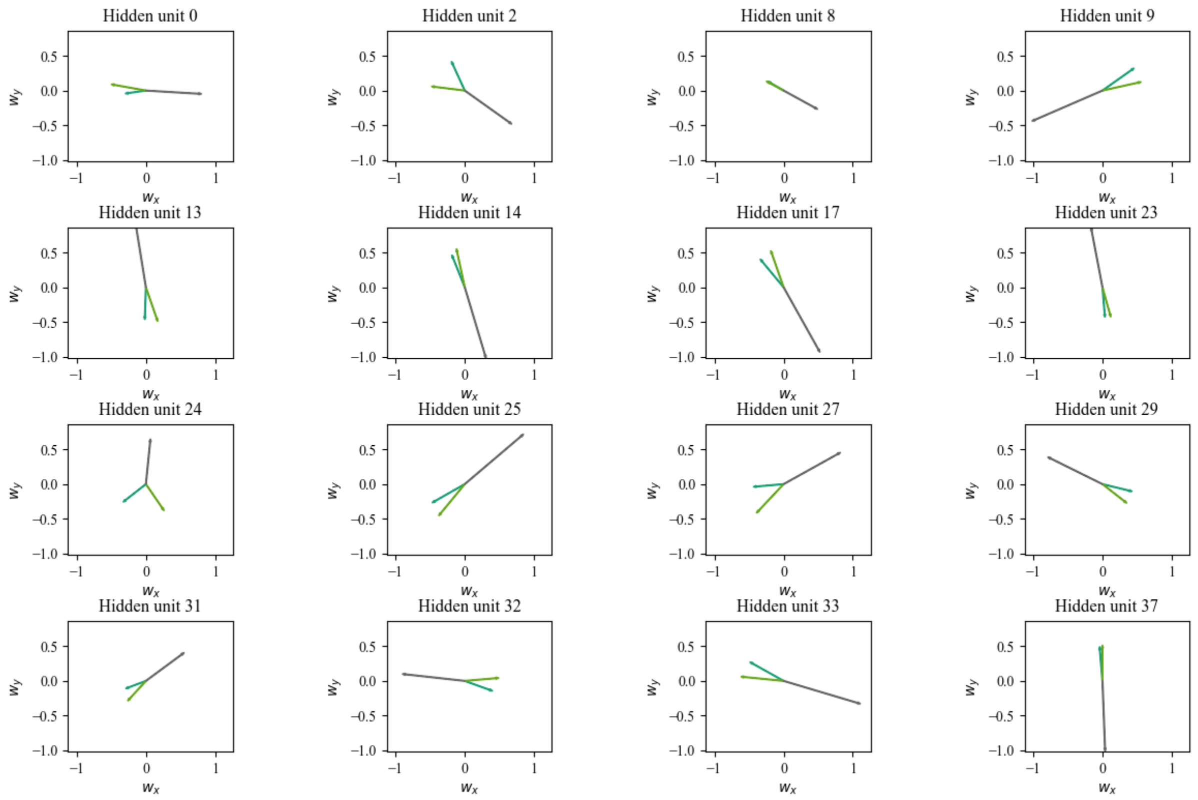

Figure SI-8: Weights from the input layer to heading hidden units, plotted as arrows in weight space. The largest vector is coloured gray, the shorter vectors are green and acquamarine.

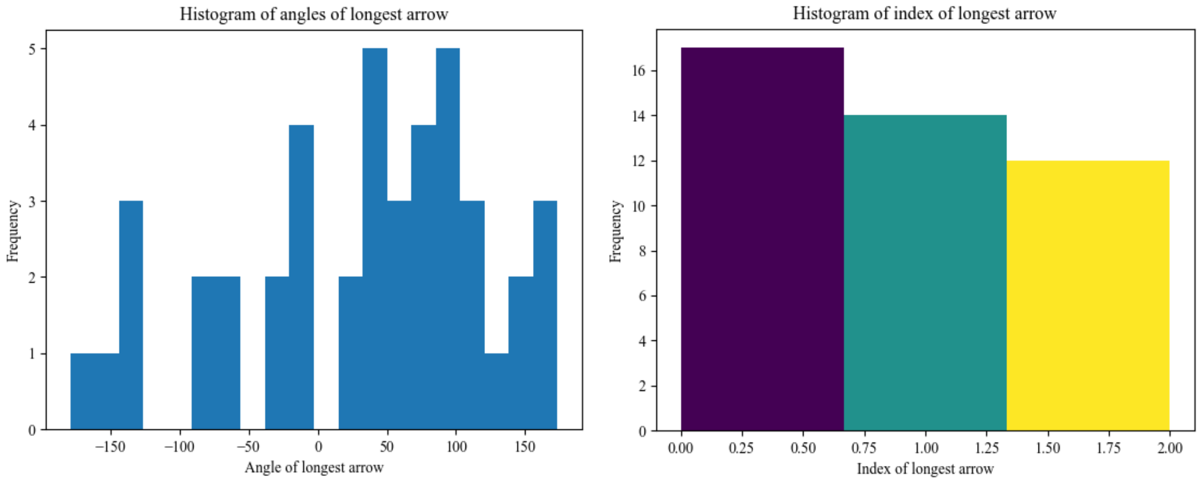

Figure SI-9: Distribution of the longest vector over all heading angles (left) and over the landmark indices (right).

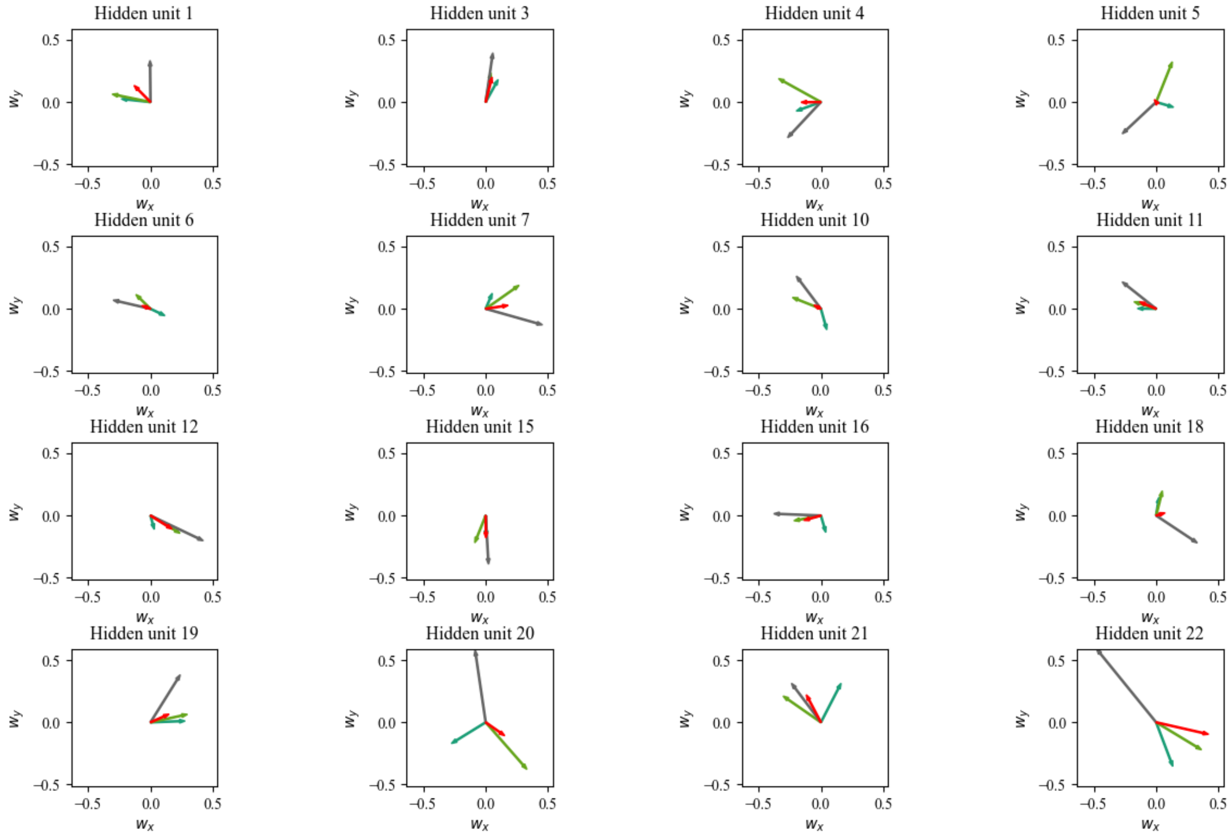

Figure SI-10: Weights from the input layer to distance hidden units, plotted as arrows in weight space. The largest vector is coloured gray, the shorter vectors are green and acquamarine. The red vector represents the weights towards the output layer.

When landmarks can no longer be uniquely identified, the task of estimating a home vector gets more difficult. If there are too few landmarks or the landmark configuration does not express a clear global orientation, the best a neural network can do is to move to the landmarks centroid. If, on the other hand, sufficient information is available ($L \geq 3$ with the landmarks in a good configuration), a neural network can point straight to the home location again. This is interesting as the neural network is denied a lot of information that will in many natural and human-made environments be available to a robot or insect.

SI-4: Tortuosity

We have increasingly degraded the information available to the neural network, removing access to a global heading and the uniqueness of landmarks. Still, the homing vector flow fields are qualitatively very different from what we observed in our simulation and real-world experiments that involve vision. Namely, most of the flow fields until now are radial flow fields, where each arrow points either straight at the home location or at the centroid landmark location. In this section, we investigate the influence of path integration drift on the nature of the flow field.

Theoretical analysis of the influence of path integration drift on home vector learning

When a robot performs path integration, its estimated position will drift over time. If the robot does not have access to a global heading cue, the robot's heading estimate will also drift over time. This is the

case for the robot used in our experiments, since it does not use a magnetometer nor does it use the time-corrected sun position.

Intuitively, if the estimated position is different from the true position, the robot's learned home vector will have an error with respect to the true home vector. Given a specific position error (e.g., 1 meter), the angular error of the homing vector will be larger when closer to the home location. If a learning flight starts at the home location and then goes further away, the largest position errors will occur furthest away as the path integration drift builds up over time. In contrast, heading drift directly leads to an angular error. For example, if the heading has drifted 5 degrees, this will directly contribute to the angular error of the supposed home vector with respect to the true home vector. The heading-based and position-based angular errors may either add up or subtract from each other.

Here we analyse a straightforward mathematical model to show that in the presence of path integration drift, the supposed home vector will be different from the true home vector. Since the supposed vector serves as a target to the neural network learning, also the neural network's direction estimate will be off. This leads to the robot moving in directions that are not straight to the home location. When the robot follows these vectors over time, it leads to a tortuous path.

For the theoretical model, we assume that the robot moves as a Dubin's car, with a constant yaw rate c and a fixed speed s. This leads to the following equations of motion:

$$\dot{\psi} = c$$

$$\dot{x} = s\cos(\psi(t))$$

$$\dot{y} = s\sin(\psi(t))$$

Given constant speed and yaw rate, these equations can be solved algebraically to give:

$$\psi(t) = ct$$

$$x(t) = x(0) + \frac{s}{c}\sin(ct)$$

$$y(t) = y(0) + \frac{s}{c}(1 - \cos(ct))$$

, where $x(0) = 0$ and $y(0) = 0$, i.e., at the home location that is the centre of the coordinate system. The angle to the home location in radians is:

$$\gamma(t) = \operatorname{atan}\big(y(t), x(t)\big) - \psi(t) - \pi$$

We now assume that the robot only has a constant yaw rate bias, b. So the robot assumes the yaw rate to be c, but in reality it is $b + c$. This leads to a difference between the estimated angle to the nest $\hat{\gamma}$ and the real angle to the nest γ:

$$e_\gamma = \hat{\gamma} - \gamma = \operatorname{atan}(\hat{y}, \hat{x}) - \hat{\psi} - \pi - \operatorname{atan}(y, x) + \psi + \pi$$

$$= \operatorname{atan}(\hat{y}, \hat{x}) - \operatorname{atan}(y, x) + \psi - \hat{\psi}$$

, in which we omitted the dependence on t for brevity of notation. This equation shows that the home angle error e_γ is the addition of the position-based error $\operatorname{atan}(\hat{y}, \hat{x}) - \operatorname{atan}(y, x)$ and heading-based error $\psi - \hat{\psi}$.

For a nonzero yaw rate bias b there are only few situations in which e_γ will be zero. First, when $t > 0$, the yaw rate bias will make $\psi \neq \hat{\psi}$. Of course, at some point the estimated yaw will be a multiple of 2π

larger or smaller than the real yaw. Hence, periodically, when $bt = 2\pi n$, with $n \in \mathbb{N}$, the heading error $\psi - \hat{\psi}$ will be zero.

However, even if this is the case, with a nonzero speed s, $\text{atan}(y, x)$ will typically be unequal to $\text{atan}(\hat{y}, \hat{x})$, except for the starting point when $n = 0$. Namely, with the equations of motion above, the robot is travelling with identical speed but along a circle of a different radius than it assumes. The position-based angular error $\text{atan}(\hat{y}, \hat{x}) - \text{atan}(y, x)$ is only zero if (x, y) and $(\hat{x}, \hat{y})$ lie on the same line from the origin. In order to make the total angular error zero, the estimated and real position should be at the same angle and be opposite of $\psi - \hat{\psi}$. Having the points on the same line is already rare, let alone with the opposite angle of the heading error.

Flow field in the presence of path integration drift

We adopted the same learning flight pattern as during the robotic and visual simulation experiments and added path integration drift. The left part of Figure SI-11 shows the presumed learning trajectory (black, dashed line) and the actual flown trajectory subject to path integration drift (grey line). The right part of Figure SI-11 shows the resulting homing vector flow field. The flow field is far more tortuous than in previous cases.

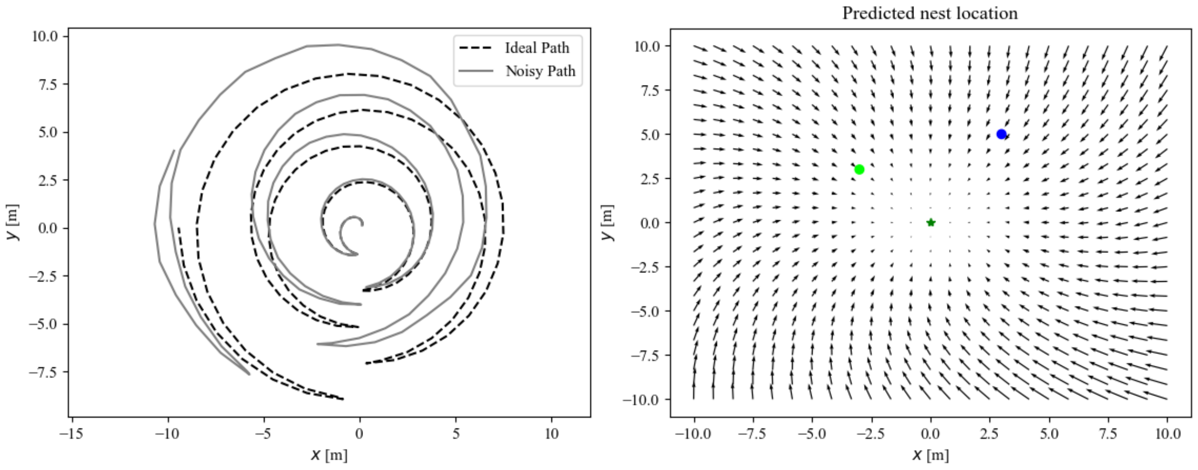

Figure SI-11: Effects of path integration noise on the homing vector flow field. *Left:* The black dashed line shows the path that the robot thinks it is flying, and which serves to generate home vector targets. The grey line shows the flown trajectory, including the path integration drift. *Right:* The resulting homing vector flow field.

Finally, we would like to remark that there may be additional causes for the non-radial flow fields experienced in visual experiments. For instance, occlusions may cause ambiguities that lead to vectors that do not point straight home.

SI-5: Verification with a visual simulator

Until now we have performed all experiments with a simplified simulator, in which the neural network has direct access to the angles and distances to the various landmarks. In a real-world application, a neural network will have to extract all information from images instead. In order to verify the findings from the above sections, we have set up an elementary visual environment in the NVidia Isaac simulator. It uses cylinders with primary colours as landmarks, and features a completely flat, green ground plane. In this environment, we used a "simple" convolutional neural network, with 2 ten-channel convolutional layers, followed by a 100-neuron hidden layer, and then a linear mapping to 2 output neurons (SI-12).

Figure SI-12 shows the results, which all verify the results from the simplified simulator in section SI-1 to SI-4. The case of the robot knowing its global heading direction is simulated by only providing images pointing North. This would be akin to the robot mentally rotating any image in the correct direction. If the robot knows its global heading direction, a single landmark suffices to pinpoint the (invisible) home location (Figure SI-12a). When the robot does not know its global heading direction (is presented with images from any direction), the best it can do is to move towards the landmark (Figure SI-12b). When the landmark is additionally placed at the edge of the learning arena, the robot would move to a circle around that landmark (Figure SI-12c). If a second landmark is added that is visually distinct from the first landmark, the robot can extract its heading from the configuration of landmarks. In that case, the network can pinpoint the home location again (Figure SI-12d). Finally, if all landmarks look the same, then the network can only pinpoint the home location when the configuration allows to retrieve the global heading direction. Indeed, the isosceles triangle in Figure SI-12e enables the network to pinpoint the home location. It must be noted here that this is a difficult visual task for the convolutional network to perform. Longer training runs were required, i.e., 100 instead of 25 epochs. Moreover, the landmark configuration had to be sufficiently distinct. For example, moving the top landmark at $(-3.5, 7.0)$ down to $(-3.5, 4.0)$ makes the task much harder, even though the triangle is still isosceles.

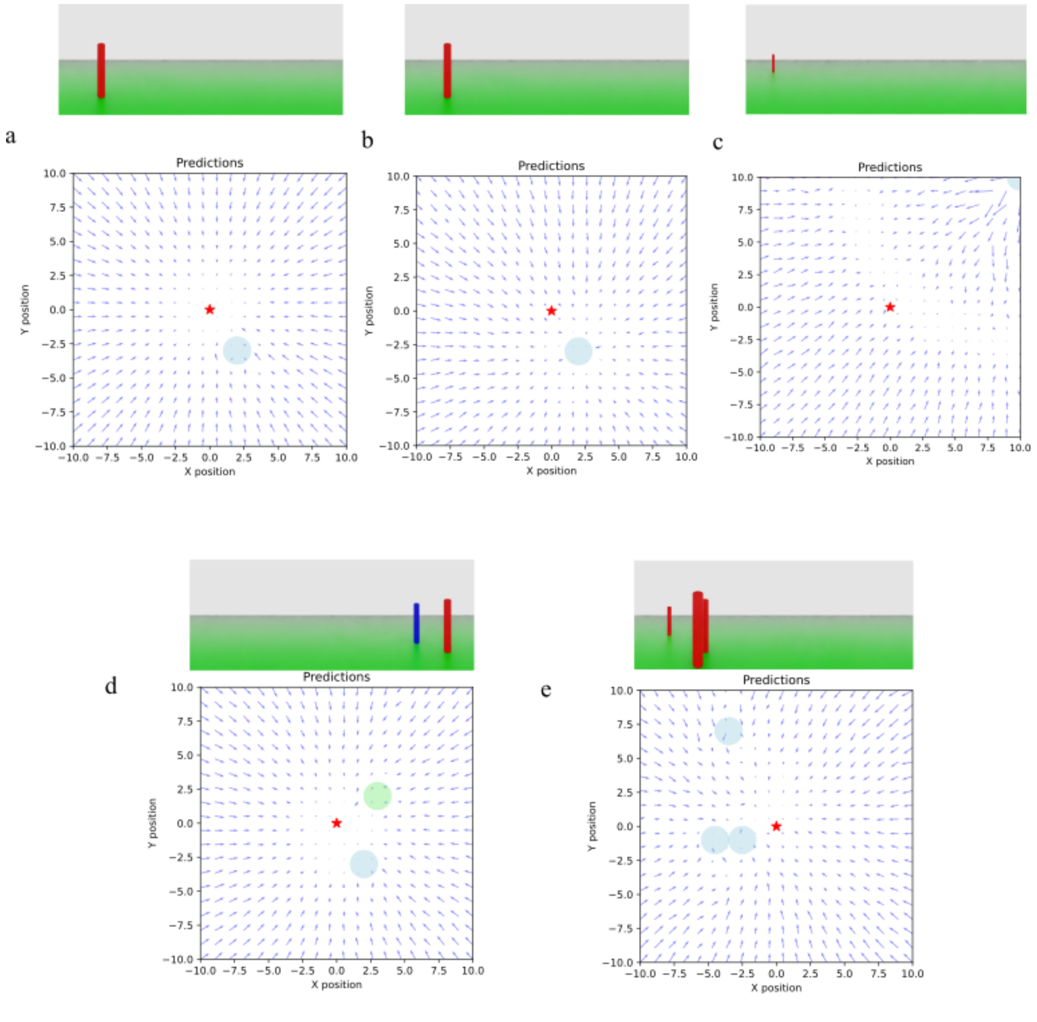

Figure SI-12: Verification of the theoretical and simulation analysis with experiments in a visual simulator. Each inset (a-e) shows the environment on the top, and a quiver plot of the trained neural network's predictions on the bottom. The home location is indicated with a red star and landmarks with semi-transparent circles.

Although the simplified visual simulator allows for many interesting additional experiments, they are out of scope for the current article. Since our main aim was to provide a robotic navigation method, we moved on to more realistic imagery, first in simulation and then in the real world. Please note that in the real world, the robot can visually recognize distant objects for extracting heading information. Moreover, a single landmark that is asymmetrically shaped can in principle also already provide heading information to the robot. The main difference between these visual cues and the sun position arises in a social context: Although local environmental cues like a large mountain or a strangely shaped tree may provide heading information to an individual robot, it would still need to align such a reference to that of other robots in its swarm. This while the sun position can be assumed to be the same for all. Future work should also delve deeper into what information is actually extracted from real-world images by trained neural networks.

SI-6: Analysis of learning flight patterns

The drone acquires training data during a learning flight conducted around a designated home location. Two primary flight patterns are considered for this purpose: a classical Archimedean spiral and a "wasp-like" pattern inspired by the nest inspection flights of wasps (*Cerceris australis*). The choice of flight pattern is critical for preventing the network from learning spurious correlations from the drone's own body, which can appear in the omnidirectional image. In a standard spiral pattern, the home location is always on the same side relative to the drone's direction of travel. Consequently, any visual artifact from the drone's hardware (e.g., an antenna) maintains a constant spatial relationship with the true home direction, creating a potential failure mode where the network learns this invalid cue instead of genuine environmental features. The wasp-like pattern mitigates this issue by incorporating back-and-forth loops, which systematically alternate the home location between the left and right sides of the drone's body, forcing the network to rely on robust environmental information.

To quantitatively compare these two patterns, we conducted a series of homing experiments in the CyberZoo, using an OptiTrack motion capture system for ground truth analysis. The experiments were performed in two distinct setups: a **feature-rich environment** populated with various objects, and a **feature-poor environment** consisting of an empty space enclosed by black curtains on three sides. For each setup, a separate neural network was trained using data from either a spiral or a wasp-like learning flight (Figure SI-13b). We then evaluated each network by initiating homing flights from eight different starting positions around the home location.

The results, shown in Figure SI-13c-e, reveal a significant performance difference in the feature-poor environment. The network trained with the wasp-like pattern achieved a mean angular error of **6.68°**, substantially outperforming the **17.23°** error from the spiral-trained network. In the feature-rich environment, this difference was less pronounced, with both patterns performing well (6.87° for spiral vs. 8.31° for wasp-like). The distance prediction error was comparable across all tests, with the spiral pattern showing a slight advantage.

Given that angular accuracy is the most critical factor for successful homing and that real-world environments can vary in feature richness, the wasp-like pattern was selected for all subsequent experiments due to its superior robustness against spurious cues. Notably, these tests also demonstrate that the system can achieve successful homing when the training data is generated using imperfect onboard odometry, as shown by the discrepancy between the odometry-based learning paths and the ground truth in Figure SI-13b. Future work could investigate the effects of a wider range of learning flight patterns, including different area sizes and sampling densities.

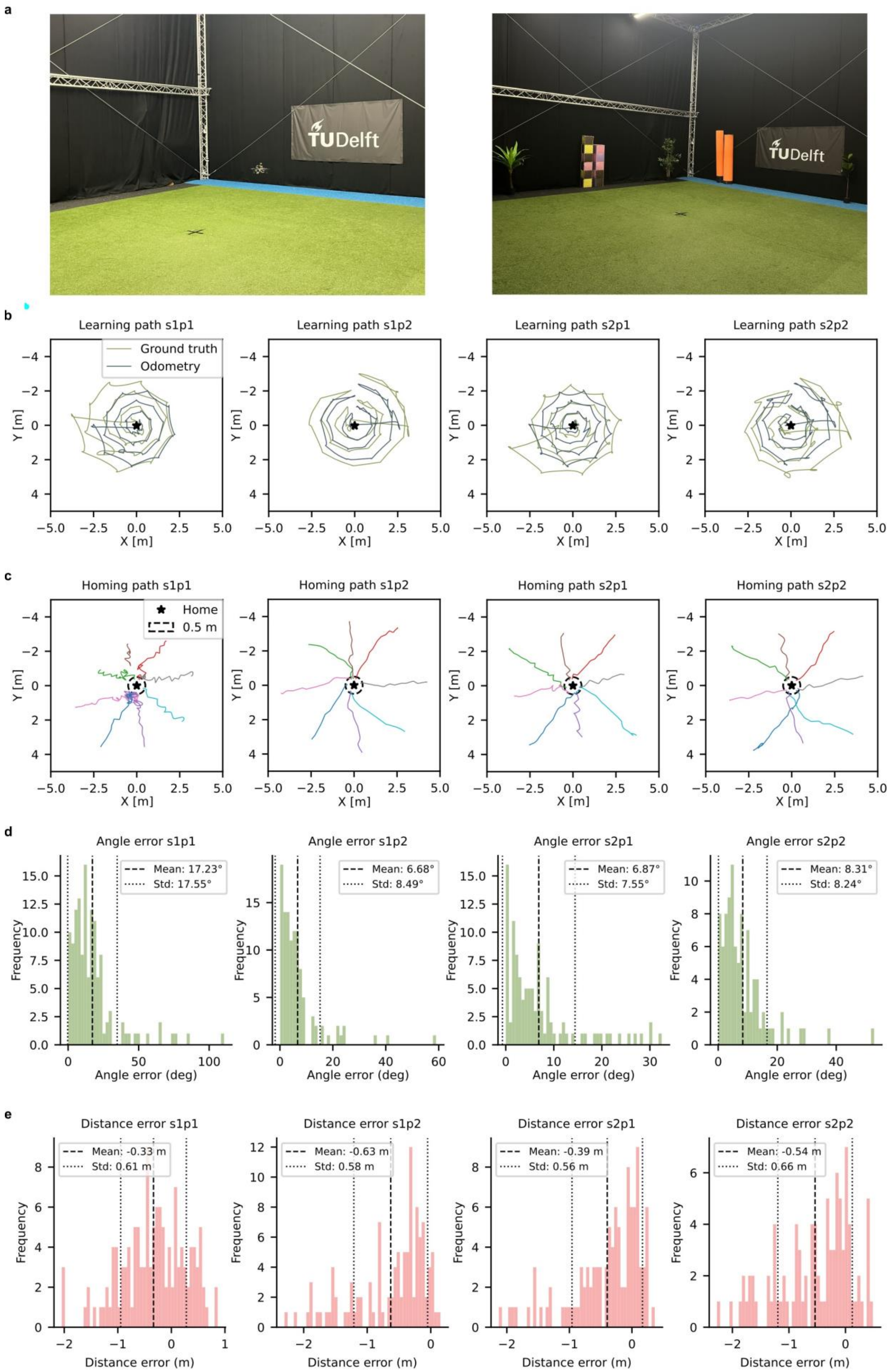
a
TUDelft
TUDelft
b
Learning path s1p1
Learning path s1p2
Learning path s2p1
Learning path s2p2
Ground truth
Odometry
Y [m]
X [m]
c
Homing path s1p1
Homing path s1p2
Homing path s2p1
Homing path s2p2
Home
0.5 m
d
Angle error s1p1
Mean: 17.23°
Std: 17.55°
Angle error s1p2
Mean: 6.68°
Std: 8.49°
Angle error s2p1
Mean: 6.87°
Std: 7.55°
Angle error s2p2
Mean: 8.31°
Std: 8.24°
Frequency
Angle error (deg)
e
Distance error s1p1
Mean: -0.33 m
Std: 0.61 m
Distance error s1p2
Mean: -0.63 m
Std: 0.58 m
Distance error s2p1
Mean: -0.39 m
Std: 0.56 m
Distance error s2p2
Mean: -0.54 m
Std: 0.66 m
Frequency
Distance error (m)

Figure SI-13: Comparison of learning patterns in feature-rich vs. feature-poor environments. a. The two experimental setups in the CyberZoo. Left: The feature-poor environment, consisting of an empty space enclosed primarily by black curtains. Right: The feature-rich environment, populated with various objects, plants, and coloured blocks to provide distinct visual cues. **b.** The two learning flight patterns (spiral and wasp-like) flown in each setup. The plots show both the ground truth trajectory from the OptiTrack system (black) and the path recorded by the drone's onboard odometry (green), highlighting the drift present in the data used for training. **c.** Homing trajectories for each of the four trained models (one for each combination of environment and learning pattern). Each plot shows eight homing flights initiated from different starting positions around the home location (black star). **d,e.** Histograms of the angular and distance errors for each condition. The results show that in the feature-poor environment, the network trained with the wasp-like pattern achieved a significantly lower mean angular error (6.68°) compared to the spiral-trained network (17.23°). In the feature-rich environment, the performance was comparable (6.87° for spiral vs. 8.31° for wasp-like), demonstrating the wasp-like pattern's superior robustness against learning spurious cues in visually challenging conditions.

SI-7 Re-analysis of honeybee data

The proposed insect-inspired navigation strategy also leads to new hypotheses on honeybee navigation. For example, based on our experimental and theoretical findings, we expect that flying based on path integration will lead to a faster, more straight flight path, whereas flying based on view memory will lead to a slower, more tortuous flight path. Furthermore, the delineation between these fly modes should be at the edge of the learned homing area (LHA).

As a preliminary verification of whether biological data is in line with these expectations, we re-analysed the honeybee trajectory data from Degen et al. (2015) (Figure SI-14a, b). By measuring the tortuosity over the inbound flights, we find that honeybees' trajectories outside of the LHA are indeed close to straight, whereas they are much more tortuous inside. This analysis was done for different scaling factors of the LHA radius (Figure SI-14c,d). The factor of 1.3 gives the highest tortuosity for visual homing, corresponding to view memory generalizing 30% beyond the learning flight trajectories. Also, the velocities of the honeybees corroborate the expected pattern of fast path-integration-based inbound flight and slower visual homing (Figure SI-14d). We provide more information on the methodology of the data re-analysis below.

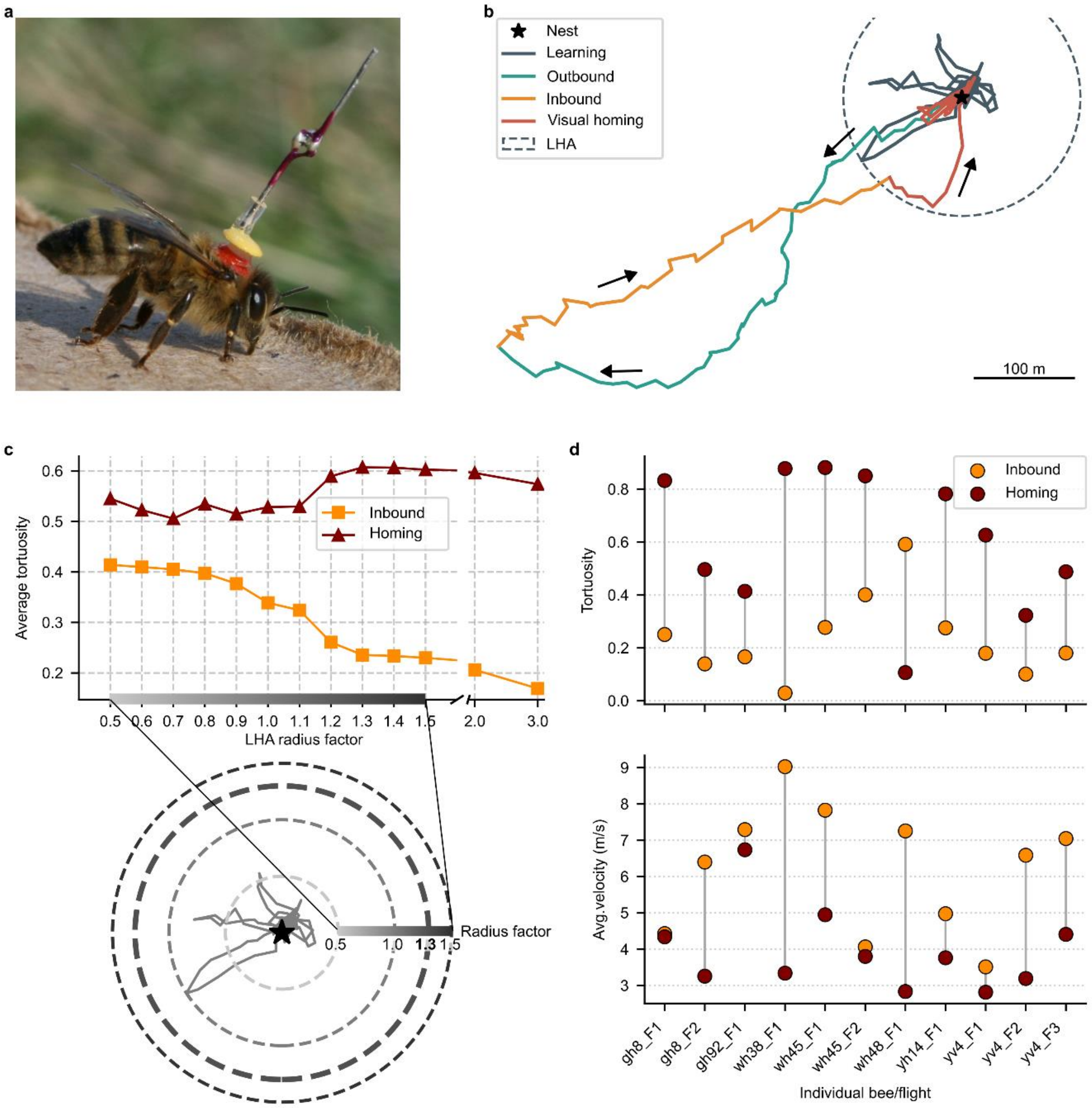

Figure SI-14: The foraging flights of honeybees reveal distinct flight characteristics aligned with the proposed navigation strategy. a. A honeybee equipped with a transponder for flight tracking, as in the original experiment by Degen et al. (2015)[1]. **b.** Representative flight paths of a single honeybee[1]. The hive is indicated by a star. The initial learning flights (dark blue) are used to define the Learned Homing Area (LHA, dashed circle). A subsequent foraging trip is categorized into three phases: the outbound phase to the furthest point (teal), the inbound phase back towards the LHA (orange), and the final visual homing phase inside the LHA (red). **c.** Average flight tortuosity for inbound and homing phases calculated using different scaling factors for the LHA radius. Tortuosity during the homing phase is consistently higher than in the outbound and inbound phases across a range of radii and reaches the highest at the LHA scaling factor of 1.3. **d.** Tortuosity (top) and average flight speed (bottom) for all eleven recorded individual foraging flights (vertical line connects the two phases of the same flight), using an LHA radius scaling factor of 1.3. For nearly all flights, tortuosity is higher and velocity is lower during the final homing phase compared to the inbound flight phase. Both differences are statistically significant ($p < 0.01$, bootstrap test).

Data overview. The dataset provided by Degen et al. (2015)[1] offers a detailed record of honeybee learning and foraging behaviour, captured using harmonic radar. The data consists of 2D flight trajectories for 115 individual bees performing 184 complete orientation (learning) flights, as well as for 20 experienced foragers performing 46 foraging flights. However, the most critical subset of this data for our analysis involves the small number of individuals for which a complete flight history—from initial learning flights through to the first few foraging flights—was recorded. Since only seven bees have this complete sequential record, our biological reanalysis focuses primarily on this specific group. A key limitation of this dataset stems from two sources of signal loss. The first is the finite range of the harmonic radar, which consistently creates a data gap at the furthest part of the flight, typically including the turnaround point. The second is intermittent signal loss, which can occur even when a bee is well within range. This happens if the bee flies too low or its body orientation is unfavorable relative to the radar antenna, and it accounts for the smaller, occasional gaps observed during the inbound and visual homing phases. When plotted, this gap appears as an artificial straight line connecting the points where the bee left and re-entered the radar's coverage.

Phase categorizing of the flights. To analyze the foraging flight behaviour, each recorded foraging trajectory was segmented into three distinct phases: outbound, inbound, and visual homing. First, for each individual bee, all of its learning flights were grouped to define a Learned Homing Area (LHA). The radius of the LHA was determined by taking the maximum distance reached across all these flights and scaling it by a factor of 1.3, a value derived from the analysis in Figure SI-14c. Each subsequent foraging flight was then categorized based on this LHA. The outbound phase is defined as the portion of the flight from its start to the furthest point reached (or to the furthest point that the radar can track). The inbound phase consists of the subsequent travel from this furthest point until the bee re-enters the LHA boundary. Finally, the visual homing phase consists of the remaining part of the flight that occurs within the LHA.

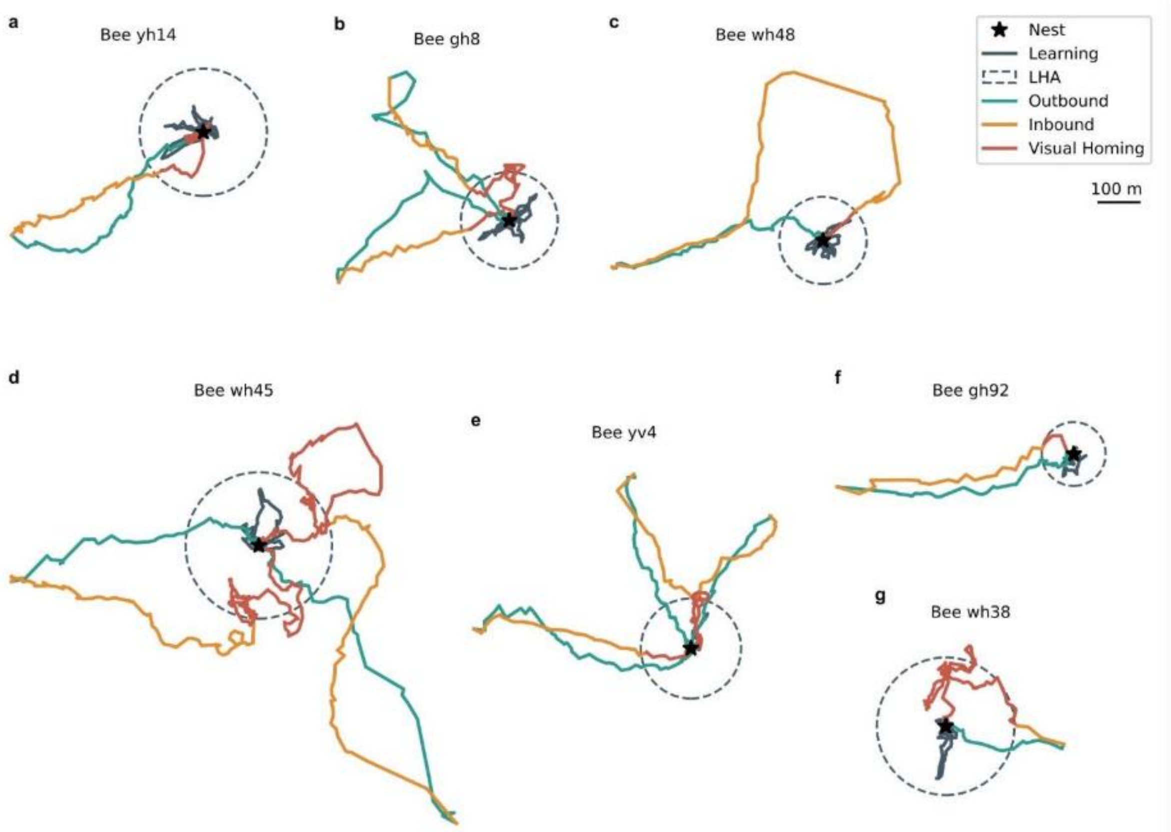

Figure SI-15: Learning and foraging flight trajectories of seven individual honeybees. The figure displays 11 foraging flights from the seven bees for which a complete, consecutive record of learning

and foraging flights was available. For each bee (a-g), all of its individual learning flights are merged and shown in grey. The Learned Homing Area (LHA), indicated by the dashed circle, is defined for each bee as a home-centered circle with a radius 1.3 times the maximum distance reached during these learning flights. This LHA boundary is then used to segment the subsequent foraging return flight into the inbound (orange) and final visual homing (red) phases, whereas the outbound phase of the foraging flight is shown in teal.

Tortuosity analysis. The tortuosity (τ), a measure of path convolutedness, is calculated for each flight phase. It is defined[2] as the ratio of the total distance travelled along the flight path (D_{path}) to the straight-line Euclidean distance between the start and end points of that phase (D_{straight}): $\tau = 1 - \frac{D_{\text{straight}}}{D_{\text{path}}}$. A value close to 0 indicates a direct, straight path, while higher values that are closer to 1 signify a more tortuous trajectory.

Velocity analysis. The average velocity for each flight phase is calculated from the discrete radar measurements, which are recorded at roughly 3-second intervals. The total path distance, D_{path}, is approximated by summing the Euclidean distances between consecutive data points. The total time elapsed, T, is the time difference between consecutive data points. The average velocity, $\bar{v}$, is then computed as: $\bar{v} = \frac{D_{path}}{T}$.

Larger dataset analysis. In the previous sections, from the entire dataset, we analyzed 11 foraging flights from seven bees, as learning flights were unavailable for the other bees[1]. The entire dataset consists of 46 foraging flights from various bees. For this larger dataset, we further performed a similar tortuosity and velocity analysis using the average LHA area defined by those seven bees. In 10 of the 46 foraging flights, the bee flew away and never returned to the nest. For the remaining 36 flights, the results are presented in Figure SI-16.

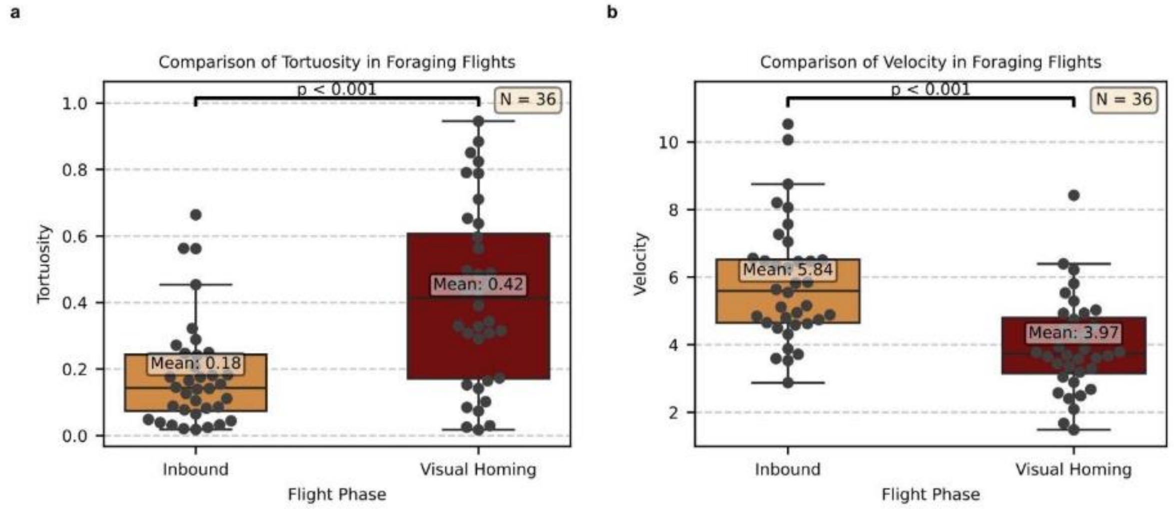

Figure SI-16: Analysis of flight characteristics in an expanded foraging dataset. This analysis uses an expanded dataset of 36 foraging flights from Degen et al. (2015) to validate the flight characteristics observed in the main text. Since individual learning flights were not available for this dataset, the Learned Homing Area (LHA) for segmenting flight phases was defined using the average LHA radius calculated from the seven bees with complete flight histories. **a.** Comparison of tortuosity between the inbound and visual homing phases. **b.** Comparison of average velocity for the same phases. Confirming the results from the main analysis, the inbound flights are significantly more direct (lower tortuosity; mean tortuosity of 0.18 vs. 0.42) and faster (mean velocity of 5.84 m/s vs. 3.97 m/s) than the final visual homing phase (p<0.001, bootstrapping test).

Bootstrapping test. To test for significant differences in tortuosity and velocity between paired inbound and homing flight phases, we used a paired bootstrap test with 10,000 repetitions. Each pair (n=11 pairs for Figure 4 and n=36 for Figure SI-16) consisted of the inbound and homing phase from a single foraging flight. This non-parametric approach tests the null hypothesis that the true mean difference between the paired measurements is zero. For each metric, we first calculated the observed mean difference from the data. We then simulated the null hypothesis by creating a distribution of these differences that was centered at zero but preserved the original variance. From this null distribution, we drew bootstrap samples with replacement to generate a distribution of 10,000 possible mean differences. A two-tailed p-value was then calculated as the proportion of simulated means that were at least as extreme as our observed mean difference. A significance level of $\alpha = 0.01$ was used for interpretation.

SI-8 Opaque and dynamic obstacles in the learning area

While the primary experiments reported in this article were conducted in an obstacle-free Learning Home Area (LHA), real-world operational environments often contain static barriers. For instance, greenhouses are frequently obstructed by support pillars and shelving. To validate the robustness of the proposed strategy under these conditions, we conducted a set of experiments in the CyberZoo with obstacles introduced into the LHA. The robot was equipped with the TF-Nova lidars described in the Methods section to enable reactive collision avoidance.

Experimental Setup

We introduced three specific obstacles into the LHA: an orange round pole (diameter of $30\ cm$), a square pole ($28 \times 28\ cm$) and one rectangular wall ($110 \times 5\ cm$). During the learning flight, the obstacle avoidance (OA, described in the main article's methods section) module actively modified the flight trajectory when necessary. Specifically, when a pre-planned image capture location was occluded by an obstacle, the OA logic navigated the robot around the blockage. If the intended waypoint is unreachable, the system captured the learning image at the robot's current location and heading. Consequently, the resulting training dataset contained positions and headings that deviated from the standard pattern. Figure 3a in the main article and Figure SI-17 below provides an overview of the experimental setup.

Homing Performance

For the homing phase, the robot was initialized at eight different starting positions and orientations locating at the edge of the arena similar to the other CyberZoo experiments, including three challenging starting states where the robot started directly behind an object (top-left, top-right, and bottom-middle in Figure SI-17). When the visual homing network provided the directional guidance, if the forward path is obstructed, the OA module overrode specific heading and movement to prevent collisions. As described in main article's methods section and can be seen in the experiments, when the direct path was blocked, the OA system forced a lateral deviation (evasion). Crucially, provided the network continued to predict a general direction toward the home.

Results

As demonstrated in Supplementary Video 5, all visual homing attempts in the cluttered environment were successful. Figure SI-18 visualizes the interaction between the homing network output and the OA module: the white squares highlight instances where the network output a correct direction vector toward home (orange arrow, Figure SI-18), but the actual executed movement deviated to bypass an obstacle (white trajectory). Subsequently, at the new position where the path is free again, the network generated a new command to proceed with homing. This confirms that the proposed visual homing network can make correct predictions even when the forward path is obstructed (e.g., front view

blocked, Figure SI-19), and can coexist effectively with a reactive avoidance layer to achieve successful homing.

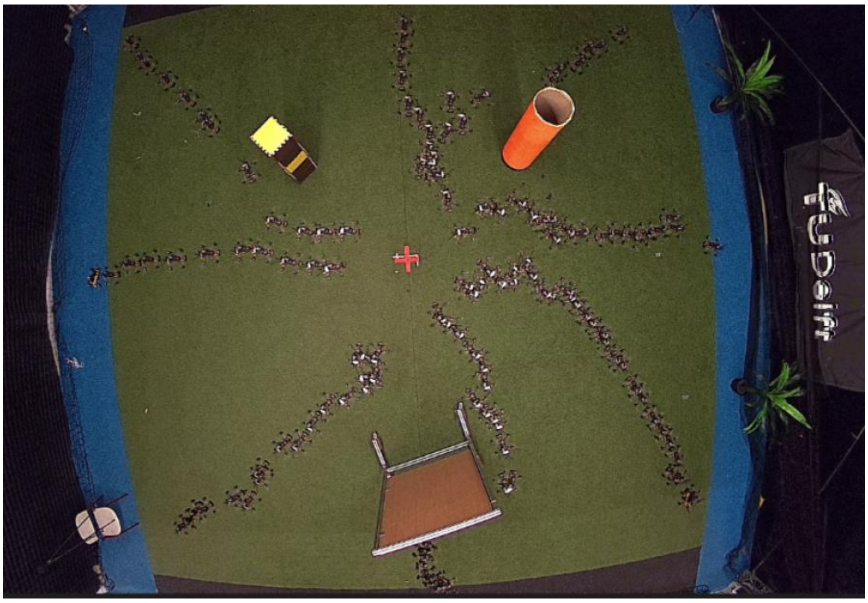

Figure SI-17: Top-view time-lapse of homing flights during obstacle avoidance experiments in the LHA. The drone takes off from eight locations around the perimeter to reach the central home position while navigating around obstacles.

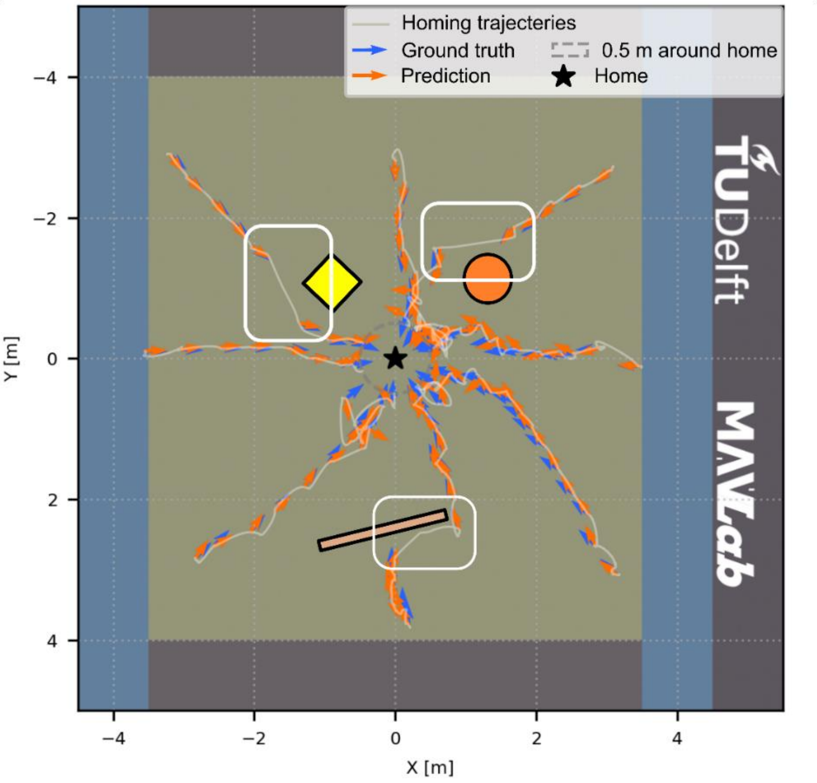

Figure SI-18: Homing trajectories and output vectors from the network recorded during experiments in the Cyberzoo with obstacles inside. Eight homing flights were performed; three of these encountered obstacles, which are highlighted by white rectangles. The predicted vectors (orange) closely match the ground truth (blue). When blocked, the obstacle avoidance module modified the path to move sideways. From this new location, the network resumed accurate predictions to reach the home position (stat).

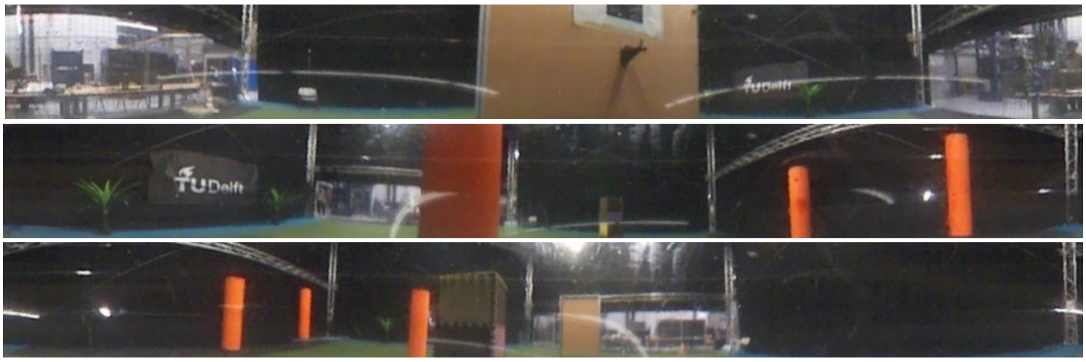

Figure SI-19: Three onboard images captured directly in front of the obstacles. Although the center of the panoramic view is blocked, the wide angle of view provides sufficient surroundings to perform homing predictions. This allows for the correct vector predictions shown in the previous figure.

Dynamic objects

Dynamic objects were encountered in various situations during the experiments. The most common cases involved experimenters and observers moving during the learning and homing phases (Figure SI-20). Additionally, in outdoor settings, environmental changes such as rapidly moving clouds occurred between the learning and homing flights.

In general, the network handles dynamic objects well, particularly when they are relatively small and inconspicuous. This robustness is largely due to the wide field of view; small dynamic elements do not significantly affect the network's overall perception. Furthermore, the supervised learning mechanism naturally teaches the network to neglect features that do not remain static throughout the learning trajectory (such as a moving experimenter), preventing them from being interpreted as critical cues. However, high-contrast dynamic elements, such as specular highlights or sun glare, can still act as dominant false landmarks. This contributes to the lower success rate in outdoor environments compared to indoor settings. For deployment in highly dynamic scenarios, such as a busy city square, future work could draw inspiration from methods that use motion detection to mask specific image regions during learning.

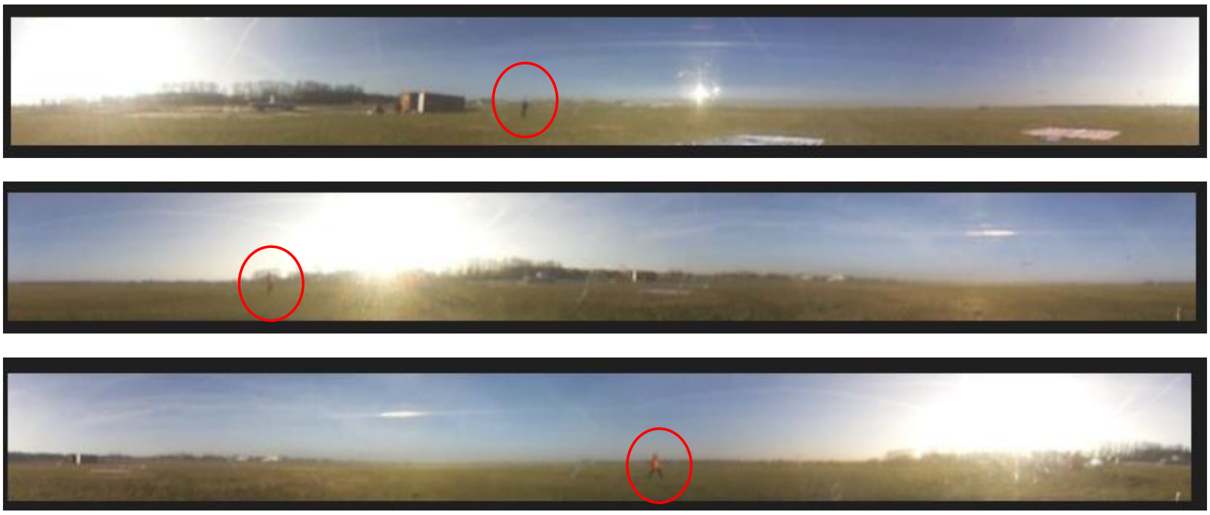

Figure SI-20: Onboard images captured during the 600-meter outdoor experiment. Red circles highlight experimenters visible in the frames. The top image is from the learning flight, where an experimenter **was** walking within the field of view. The middle and bottom images **show** a different experimenter changing locations during the homing phase.

SI-9 ORB-SLAM 2 in one of the experiment environments

The main state-of-the-art approach to robot navigation, Simultaneous Localization And Mapping (SLAM), relies on the construction of a map of the environment. As discussed in the main article, SLAM allows a robot to travel from any place on the map to any other place that is also on the map. Moreover, if the map contains information on all obstacles in the environment, this path can be planned to be optimal in terms of travel distance.

The proposed, bee-inspired navigation strategy is different from SLAM, as it only allows robots to travel from and to a central home location. In other words, it permits a strict subset of SLAM's navigation capabilities. Of course, this means that SLAM could also be used for this task. Hence, we have also performed an experiment in which we investigate how much memory a conventional SLAM algorithm would use in the same environment.

To this end, we ran a popular SLAM method[3], ORB-SLAM 2. Although ORB-SLAM 2 was released already in 2017, it is still one of the most popular methods due to its computational efficiency and robustness[4,5]. Because it was not designed for low-resolution omnidirectional images, we apply it to a higher-resolution forward-looking camera (which we mounted on our robot solely for this purpose). The forward-looking camera recorded video at 30 FPS with a 640×480 pixel resolution. Because our interest here is only in the memory consumption of the map, the ORB-SLAM 2 experiments only involve the mapping phase, not any autonomous navigation.

We executed ORB-SLAM2 within a Docker container on a desktop computer equipped with a Intel® Core™ i9-11900K @ 3.50GHz × 16 processor. The camera was calibrated using a checkerboard pattern and the OpenCV library to obtain the intrinsic parameters. The recorded video was pre-processed to the required format, similar to the opensource TUM Mono SLAM dataset. To ensure stable feature tracking across sequences, the number of feature points was set to 10,000. System performance was quantified by monitoring the resource utilization of the Docker container using the docker stats command. We recorded and plotted the computational load across three distinct stages: idle (pre-execution), system initialization, and the active tracking and mapping phase. The resulting performance profile is illustrated in Figure SI-21.

The memory consumption grows from ~600 MB at initialization to ~1200MB after constructing the map of the environment. Hence, discounting the memory used for the supporting software (forming the memory at initialization), the map takes up ~600 MB of memory. Please note that the CPU usage of this rather efficient SLAM method is over 200%, saturating two of the computer's processors.

In the discussion of the main article, we compare the memory used by the SLAM map with the memory taken up by the larger attention neural network. Directly comparing these results in a factor $\frac{600000 \text{ kB}}{42.3 \text{ kB}} \approx 14184$, i.e., four orders of magnitude. At inference time, the neural network also needs an input image, which takes up ~90kB in case of our robot. Counting also the image, the factor becomes: $\frac{600000}{132.3} \approx 4535$, i.e., three orders of magnitude. This is the factor mentioned in the discussion when comparing Bee-Nav's view memory network with high-resolution SLAM maps.

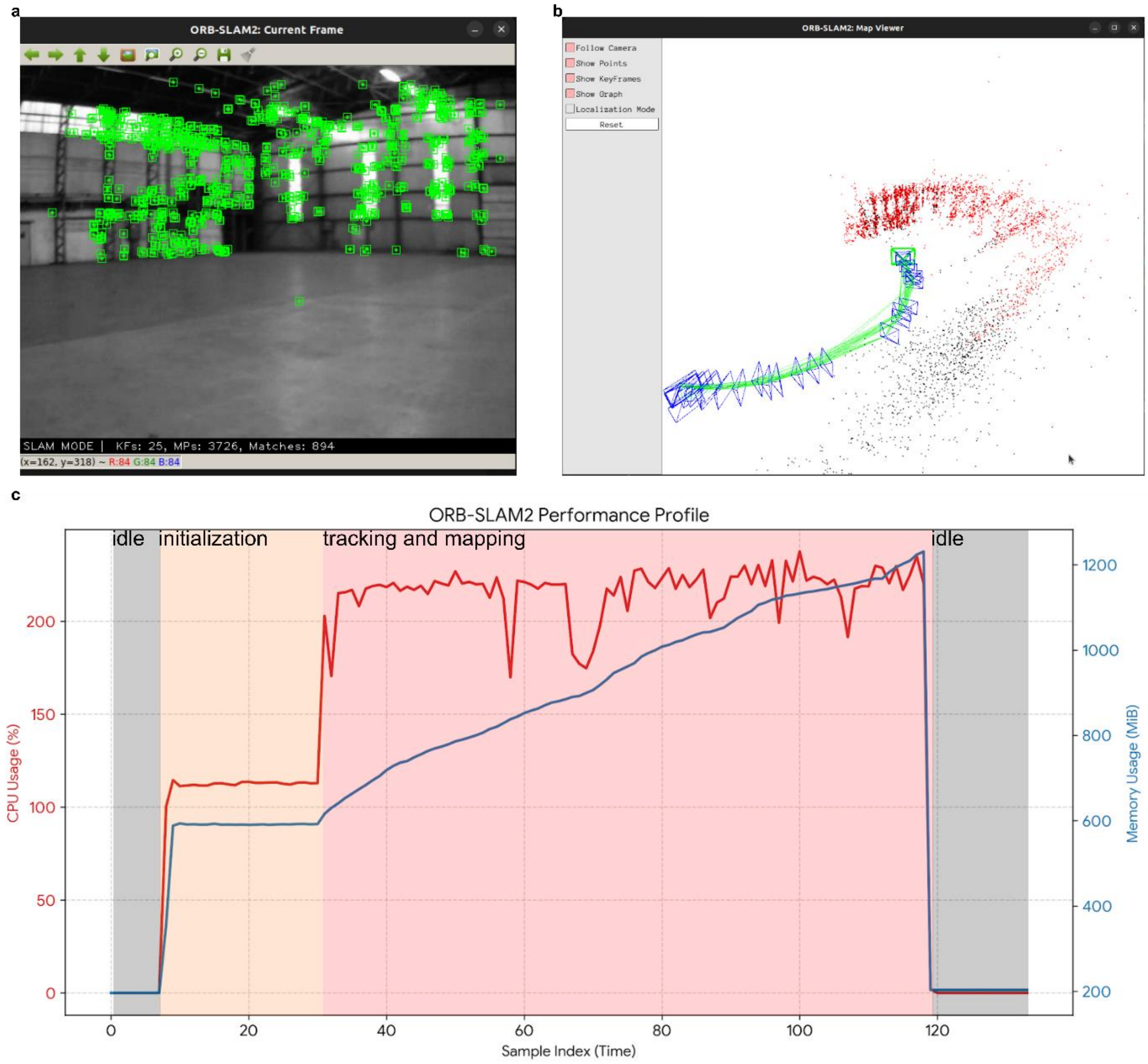

Figure SI-21: a. One of the frames show SLAM was performing feature extraction and matching in the Unmanned Valley Valkenburg (UVV) indoor hangar test area. **b.** The trajectory and key-frame features that it stored. **c.** CPU and memory usage during SLAM operation. Gray shaded areas indicate phases where SLAM is inactive. The light pink area indicates the SLAM initialization phase, where the CPU is active but memory usage remains constant. The dark pink area shows the tracking and mapping phase, during which memory usage grows linearly with the distance travelled. The CPU used was an Intel® Core™ i9-11900K @ 3.50GHz × 16.

SI-10 Visual simulation environment

In this section we provide some additional information on the visual environment of the simulation experiments. These experiments were performed in automatically generated forests. The environment generation script selected the trees randomly from the standard set of trees available in the ISAAC simulation ecosystem.

Below, we show twenty-five example trees, placed on a 5×5 grid in a 20×20m area from the 3D object set on a white surface. It can be seen that the trees are generally quite visually distinct. Hence, we expect that the network can identify many of these trees as individual and unique landmarks (e.g., by means of the distinct color or shape). Please note that the visual distinctiveness of the trees suggests that the case faced by the neural network in the visual simulator is more like the theoretical case in

which each landmark has its own identity than the case in which each landmark is identical (referring to the analysis in SI-1-5).

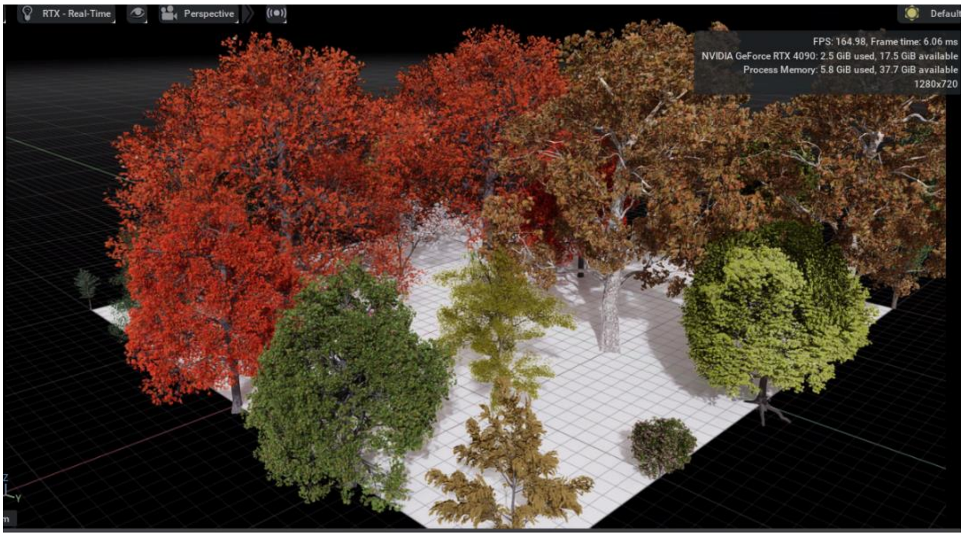

Figure SI-22: randomly sampled trees from the Omniverse tree assets.

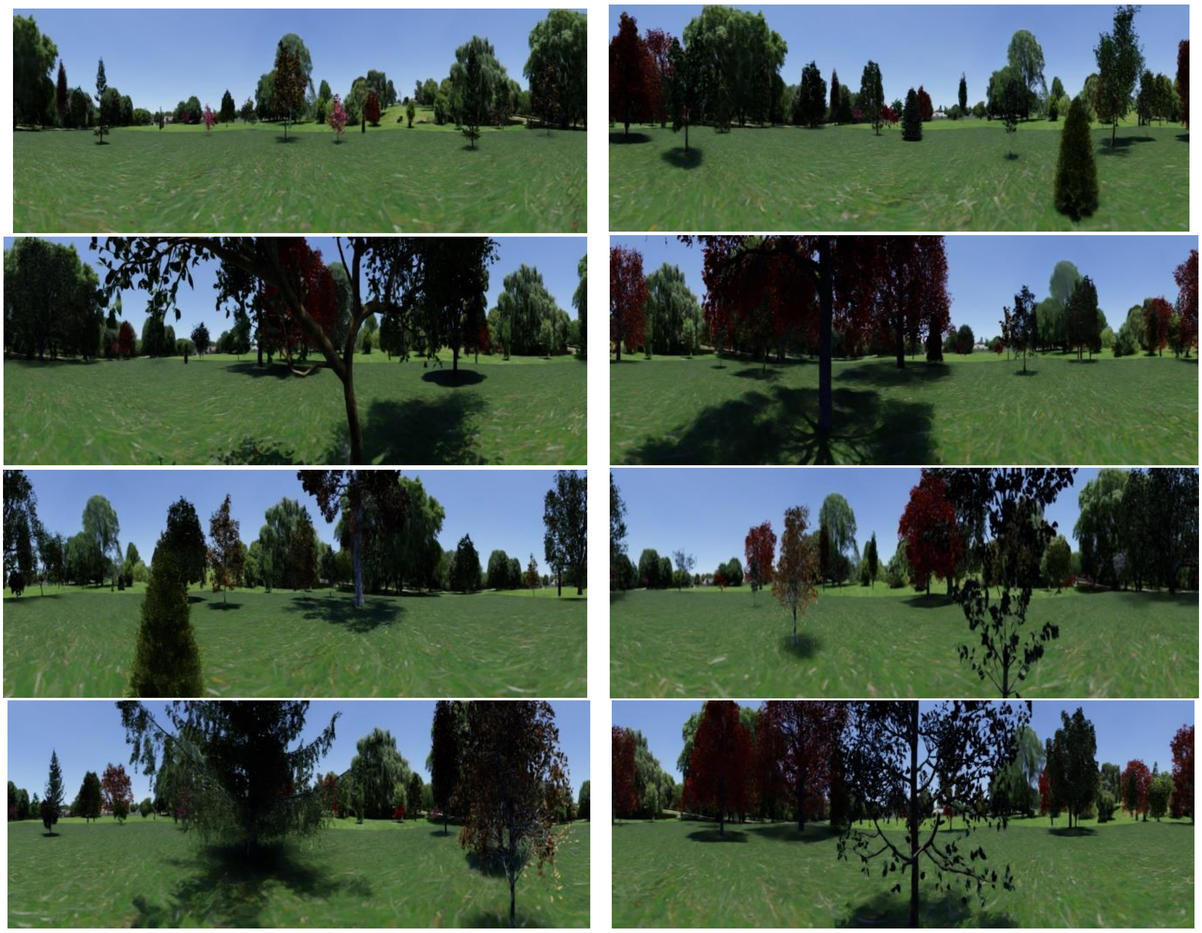

Figure SI-23: Example omnidirectional images from differently generated virtual forests.

SI-11 Path integration drift characterization

As explained in the Methods section of the main article, we use a Gaussian noise model for path integration drift. First, we performed path integration experiments in our flight arena, equipped with a motion tracking system (ten Optitrack Prime 17W motion capture cameras). Subsequently, we tuned the parameters of the model to fit with the data from the path integration experiments. Moreover, we fit the model parameters to path integration data reported in the literature, both for robots and insects.

Path integration experiments

We performed various path integration experiments with our drone, measuring the position and yaw drift over time. In order to cover substantial distances in our flight arena, we had the drone continuously fly a square pattern of $4 \times 4\ m$. We did this in three different conditions:

- **Condition 1:** Artificial grass floor without wind, characteristic of most indoor experiments.
- **Condition 2:** A highly textured ground, with sufficient cues for the optical flow sensor.
- **Condition 3:** Artificial grass floor with wind, resembling typical challenges during outdoor experiments.

The experiments provided data for position drift after different flight distances and yaw drift at given flight times. For each setup, we performed five flights to generate statistics.

An overview and one example flight for each scene setup are shown in Figures SI-24-26, with final statistical results presented in Figure SI-27. As expected, path integration performs best in the texture-rich environment (condition 2), and worst in the grass setting with wind (condition 3). It is worth noting that actual path integration performance relies heavily on conditions such as sensor calibration, initialization, vibration (propeller states), temperature changes, and the path and movement during the flight. This sensitivity also explains some of the wide spread of errors seen in Figure SI-27.

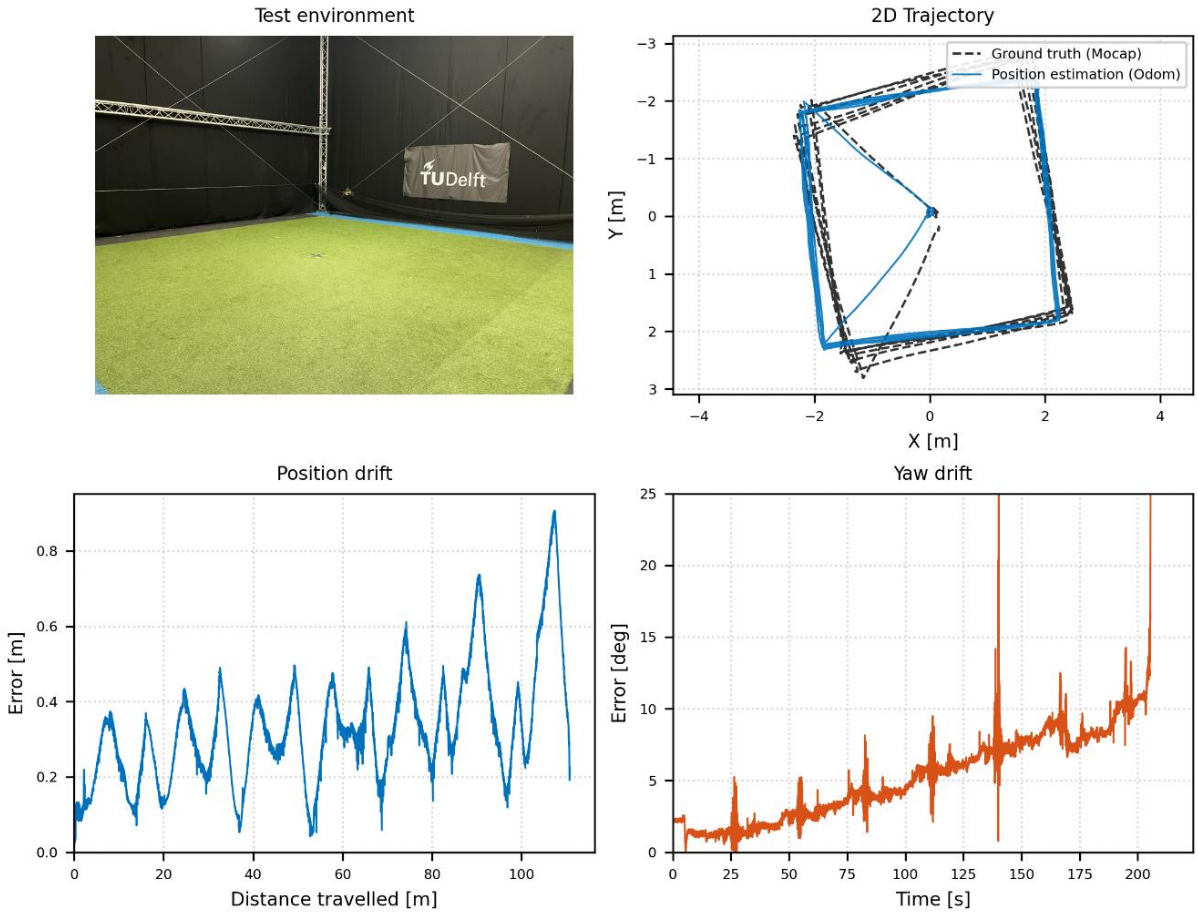

Figure SI-24: Example results of the robotic experiment for measuring path integration drift in our motion tracking arena in condition 1. *Top left:* The test environment, with artificial grass, so limited texture. *Top right:* Motion capture ground truth position vs. odometry-based position estimate. *Bottom left:* Position error over flown distance. *Bottom right:* Yaw drift over time.

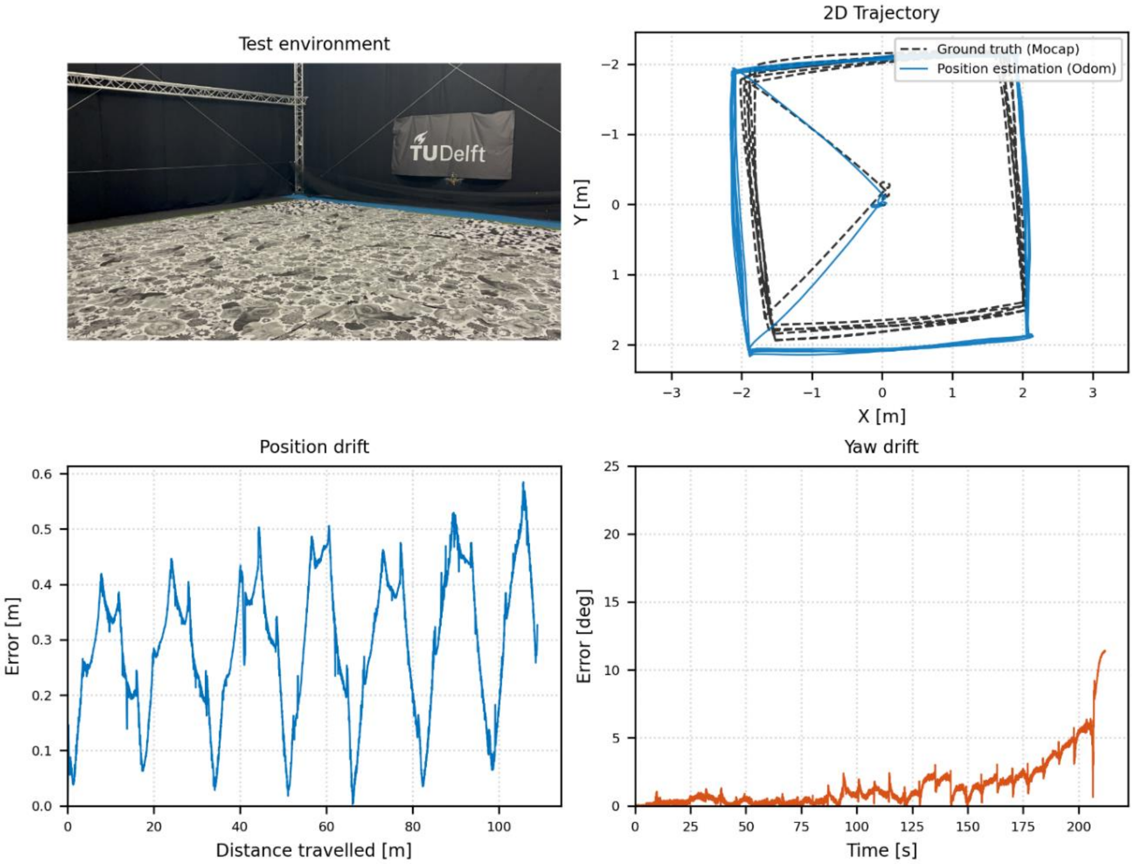

Figure SI-25: Example results of the robotic experiment for measuring path integration drift in our motion tracking arena in condition 2. *Top left:* The test environment, with rich textures on the ground. *Top right:* Motion capture ground truth position vs. odometry-based position estimate. *Bottom left:* Position error over flown distance. *Bottom right:* Yaw drift over time.

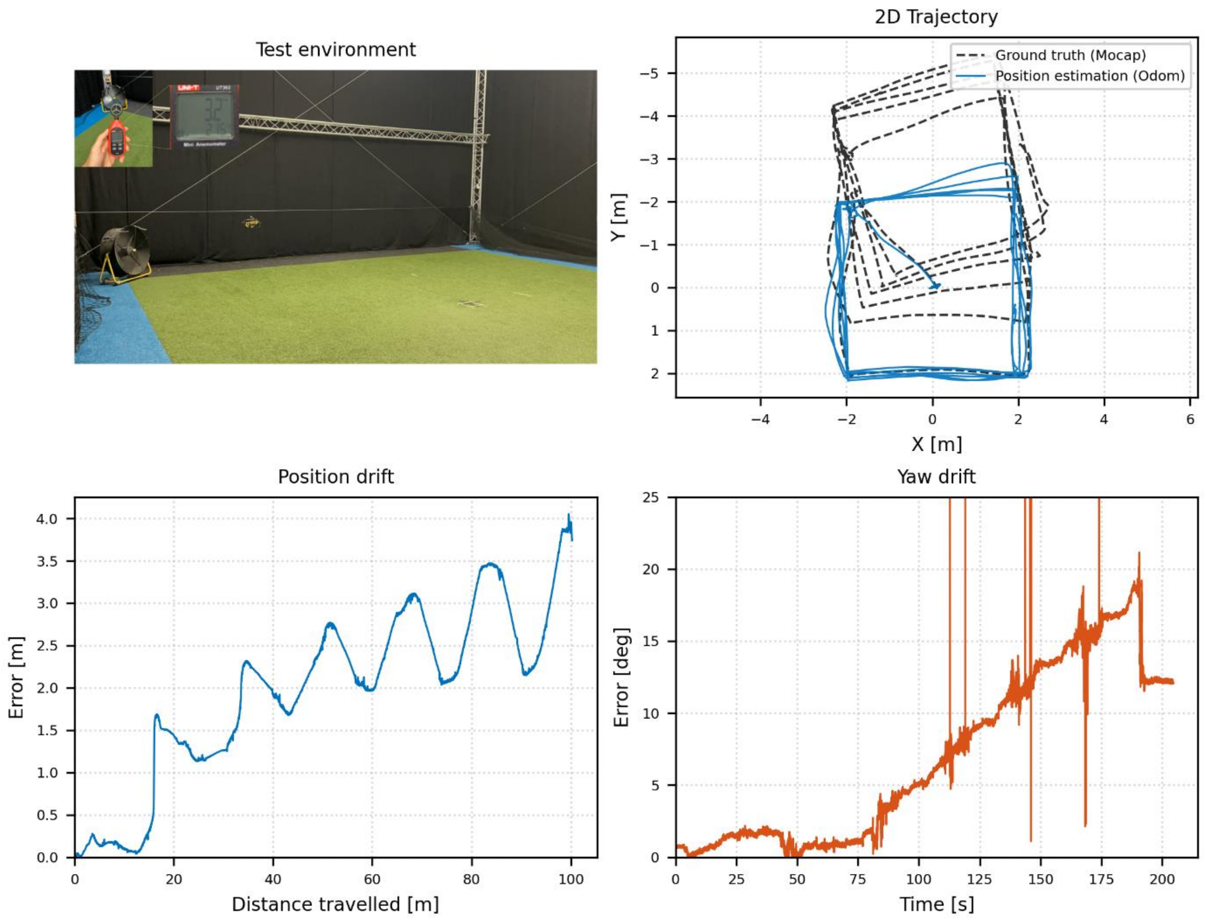

Figure SI-26: Example results of the robotic experiment for measuring path integration drift in our motion tracking arena condition 3. *Top left:* The test environment, with artificial grass, so limited texture, and a fan located at the corner with peak wind speed of 3.2 m/s. *Top right:* Motion capture ground truth position vs. odometry-based position estimate. *Bottom left:* Position error over flown distance. *Bottom right:* Yaw drift over time.

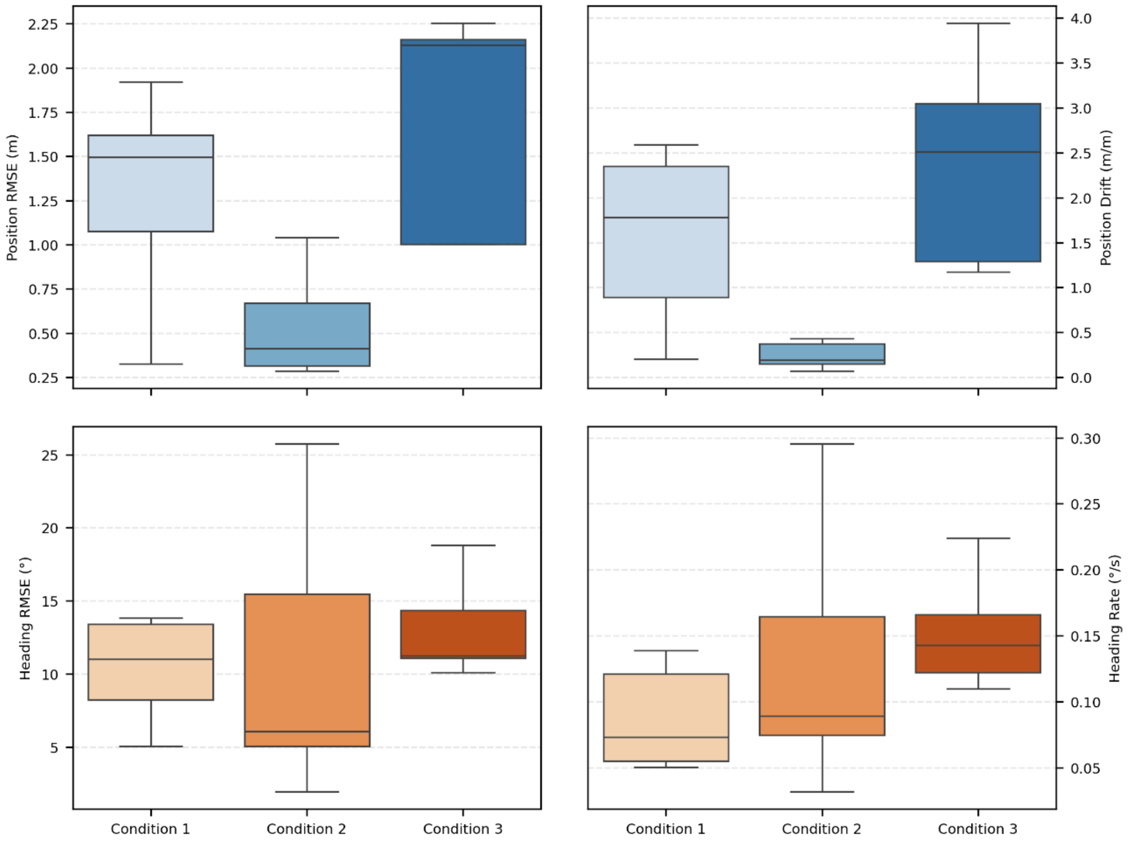

Figure SI-27: Top row: position error. Bottom row: heading error. The left panels show the RMSE error for data points recorded during flight across different environments. The right panels show the error drift rate (normalized by travelled distance for position, and by time for heading). The drift rate is calculated as the final accumulated drift divided by the total travelled distance (top) or flight duration (bottom).

Noise parameters

After the experiments, we searched for noise parameters with which the Gaussian noise model approximated the robot's drift statistics. For this we chose the data from the experiment with limited grass texture, as it represents an intermediate case. In simulation, we had a simulated drone flying squares of the same size as the real robot. We manually tuned the noise parameters, so that the path integration drift of the model approximated that from the real experiments, in which the position drift was ~0.4m at 100m and ~5° after 100s. We finally converged on the settings of $\sigma_\psi = 0.63°$, and $\sigma_d = 0.10$m for our robotic system, which – averaged over a 1000 simulated square-shaped flights – gave a mean yaw drift after 100s of 5.03° and a mean position drift after 100m of 0.91m. One example trajectory from the simulated drone is shown in Figure SI-28.

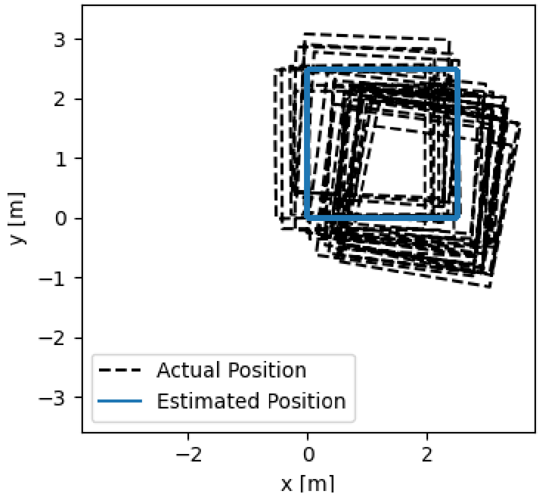

Figure SI-28: Actual (black dashed) and estimated (blue, solid) trajectory of a simulated drone trying to fly a square trajectory for 120 seconds, while experiencing path integration drift.

With these settings, we simulated a 1000 outbound block-shaped search travels and straight inbound travels. This resulted in a Learned Home Area (LHA) that is 3.84% of the total flight area. We then repeated the simulation-based "noise level" experiment, scaling both σ_ψ and σ_d with $\{0.10, 0.5, 0.75, 1.0, 1.25, 1.5\}$. With a requirement of the LHA containing 99% of the return positions, this gives LHAs that span from 0.1% to 10.82% of the total flight area.

The path integration used by the drone in the real-world experiments is quite crude. As explained in the Method section, it uses PX4's visual odometry estimate from a downward looking camera and laser. For the yaw estimation, the Extended Kalman Filter depends on noisy MEMS gyro measurements, which are integrated over time. Importantly, it does not feature a heading measurement (no "compass"). Path integration without a compass of any sorts is referred to as "idiothetic" path integration in the biology literature[6], and is known to be relatively inaccurate.

In order to give a more comprehensive idea of what could be achieved with robotic systems, we also fitted our noise model to what is known of two methods from the literature. First, more advanced versions of Visual Inertial Odometry (VIO), using a frontal camera and IMU, feature a better path integration accuracy. To illustrate this, we also determined parameters for the noise model that led to a similar drift as that of the visual-inertial odometry method "SVO-GTSAM", which was reported to have a mean drift of ~0.3m and yaw drift of ~0.8° after 35 meters of flight[7]. Our noise model, with $\sigma_\psi = 0.25°$, and $\sigma_d = 0.015$m leads to similar statistics: a mean position drift after 35m of 0.32m and mean yaw drift of 0.79°. With this VIO, the LHA percentage of the total flight area would be 0.74% according to the simulation experiments. It has to be noted that SVO-GTSAM requires substantial processing. Hence, it would require considerable onboard computational and memory resources – which goes against the efficiency of the proposed navigation method.

Second, we also fit the noise model to a robotic odometry method by Stankiewicz and Webb[8] that does use a compass, in the form of a magnetometer. They use an efficient, bio-inspired VIO method and report a position drift of 1.5m after 100m of robotic flight. The compass version of the noise model (see Methods), with $\sigma_\psi = 5.5°$, and $\sigma_d = 0.15$m also leads to 1.5m drift after 100m of travel. The corresponding percentage of the LHA is 0.24%. This example demonstrates that sensing of an absolute yaw angle (called "allothetic" path integration in the biological literature[6]) substantially improves the accuracy of path integration and hence the efficiency of the proposed navigation strategy.

Insect path integration accuracy

Determining path integration accuracy for real insects is challenging[6]. For ants, a Gaussian noise model for path integration has been proposed[9] that was fit to data from ants[10]. In that latter study, ants were captured 10 meters away from the nest and displaced in darkness to a different site. After displacement, the ants would first use their path integration estimate to go back to the nest location. However, because the other site did not have the nest, they would commence a search upon not detecting the nest at the expected location. We essentially use the model from[9] for the case of allothetic path integration. Translating the settings from that article to the standard deviation used in our model, we arrive at $\sigma_\psi = 57°$, and $\sigma_d = 0.38$m for the simulations. Note that although the angular noise seems large, it does not accumulate over time. This setting leads to an LHA percentage of 7.6%.

We have searched for a similar model for honeybees, but were not able to find any. However, recent honeybee experiments[11] form an indication of the path integration accuracy. In particular, the researchers tracked honeybees that were recruited to go to different foraging locations for the first time. They were then able to measure where the honeybees would start searching. Notably, for a foraging site 2300m away from their "hive 2", the mode of the search location distribution was only ~200m away. Although there are many factors that can contribute to the inaccuracy here (including "noise" in the communication of the honeybee dance), this offset can be regarded as an indicator for the path integration drift. As an approximation of honeybee odometry accuracy, we have fitted the allothetic noise model to the data for the furthest foraging site, F6 (figure 6 in the original article[11]). We selected this site because larger distances lead to more reliable parameter estimates than smaller distances. The best fit was achieved with settings of $\sigma_\psi = 34.5°$, and $\sigma_d = 0.38$m. This leads to 201.1m drift after 2300m of straight flight, and an LHA percentage of 3.4%.

Finally, please note that the Gaussian noise model represents only a coarse approximation to the modelled systems. The model does not directly capture the physical processes underlying path integration drift in the modelled systems, whether it concerns the drone used in the real-world experiments or the ants and honeybees in this subsection. Measurement errors in these systems are likely not Gaussian, and also depend on the circumstances, such as the properties of visual texture, wind, light conditions, electro-magnetic disturbances, clouds, etc. More detailed models should be investigated in order to obtain more accurate path integration drift estimates. However, we expect the main observations resulting from the analysis to hold. First, thanks to the combination with path integration, view memory can be limited to a relatively small area. Second, path integration with a compass substantially reduces the area that needs to be learned.

SI-12 Comparison between Bee-Nav and a mushroom-body-inspired approach in the same environment

To compare the performance of our proposed method, we implemented a recently published Mushroom Body (MB) homing model[12]. We conducted similar homing experiments using both our compact network model and the MB homing model within the photo-realistic visual simulator across two distinct environments.

Mushroom Body Implementation

We utilized the source code from Gattaux et al.[13] for the fisheye image-to-Projection Neuron (PN) projection and the Mushroom Body (MB) architecture, with slight modifications. Consistent with the description in Gattaux et al., the network consists of two mushroom bodies, each containing two output neurons (MBONs) representing the left and right familiarity scores. The parameters for the model were set to 50,000 Kenyon Cells (KCs) with a connectivity of 16 synapses per KC, and a sparsity constraint retaining the top 0.01 (1%) most active KCs.

Training Phase

Both models were trained using the same 'wasp-like' learning flight pattern covering a radius of 6.5 m around the home position. This pattern ensures that the captured views of the home are located to the left or right of the current view, which is a key requirement for the MB homing model. The main difference lies in the sampling density: the compact model used fewer samples (56) compared to the MB model (560). This difference was intended to reproduce the high frequency of image capture during the learning phase described in the original MB model article.

During training for the MB model, each image was associated with a label indicating whether the home location was to its left or right. Based on this label, the corresponding KC-to-MBON connections were suppressed (simulating anti-Hebbian learning) to encode the home direction. Conversely, the compact model was trained using the parameters described in the 'Methods: Visually realistic simulator' section.

All training and homing images were processed into fisheye representations, filtered using a Sobel filter, and projected onto projection neurons using code adapted from[13]. The pipeline for this image processing is illustrated in Figure SI-29.

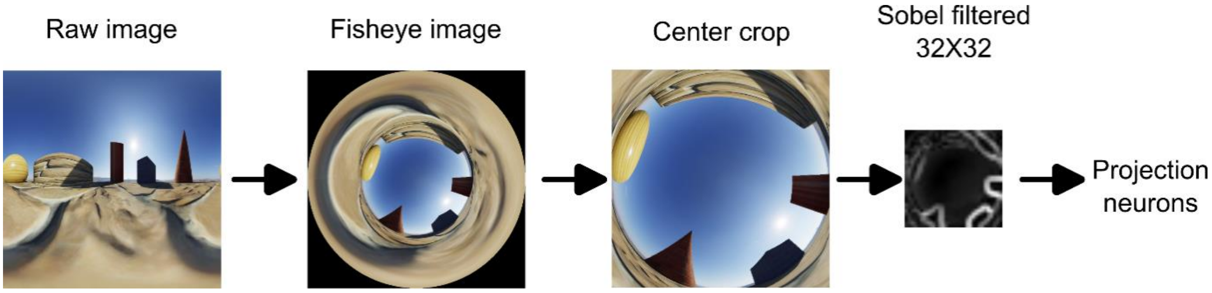

Figure SI-29: Image processing pipeline for the mushroom body-inspired (MB) homing model. The raw image generated in the simulator is first warped into a fisheye view, after which a large part of the ground is cropped out. The image is then processed by a Sobel filter and finally converted into 1D projection neurons.

Homing Procedure

For the testing phase, the agent was initialized at 16 different locations, each 6-6.5m (the radius of the learning flight) away from the home position, with varying initial headings.

For MB model, navigation was controlled by calculating the difference between the left and right Output Neuron (MBON) activities of each mushroom body. We applied a proportional gain to this difference to determine the turning angle, followed by a stepwise forward movement of 0.2 m. For our compact model, homing was achieved using the setup described in the "Methods: Visually realistic simulator" section.

For both models, the stop conditions were defined as:

1. **Success:** Reaching within a 0.5m radius of the home.
2. **Failure/Timeout:** Exceeding a maximum of 500 steps.

Environments

We evaluated learning and homing performance across two distinct environments:

1. **Forest environment:** The environment featuring 15 trees distributed across the area, which is similar to the environment used in the main article.
2. **Geometric environment:** A less cluttered environment containing simple geometric shapes around the horizon.

Results

The experimental results are presented in Figure SI-30. In the Geometric Environment (left column)—which was designed to resemble the open environments with distinct landmarks used in[13] — the MB model achieves a high success rate. Notably, the resulting trajectories exhibit significant meandering, closely replicating the behavior observed in the robotic experiments of the original study. This serves as a verification of our MB implementation. In contrast, our compact model demonstrates robust performance in the same environment, maintaining highly efficient, straight paths toward the goal.

In the Forest Environment (right column), the performance of the MB model degrades. Despite our attempts to adapt the model—such as increasing the number of Kenyon Cells (KCs) from 5,000 to 50,000 to handle the increased visual load—the strategy struggled with the ample texture and visual clutter, often leading to navigation failures where agents became trapped in local minima. Conversely, the compact network maintains consistent, robust performance, achieving homing precision and path efficiency comparable to its results in the geometric environment.

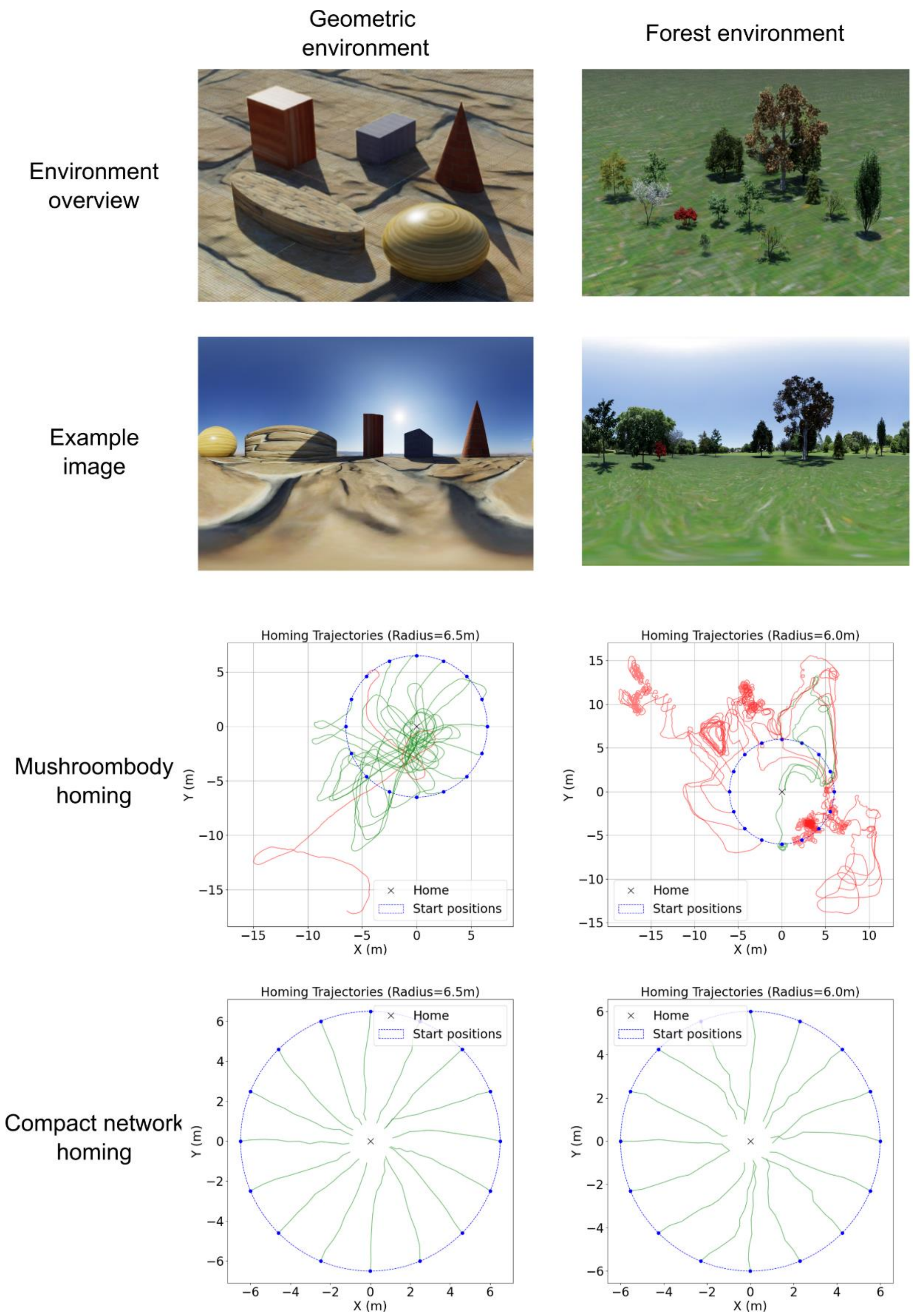

Figure SI-30: Comparative homing trajectories in simulated environments. From top to bottom: isometric view of the experimental environment; an example of the raw image; Mushroom Body (MB) model homing trajectories starting from 16 different locations and headings; and compact network homing trajectories. Green indicates successful homing, while red indicates failure. Left: In the geometric environment, the MB model reaches home but exhibits high path tortuosity, whereas the compact network (ours) produces direct paths. Right: In the forest environment, the MB model suffers

from local convergence failures. The compact network maintains robust performance across both environments.

MNIST classification benchmark

In order to get insight into the representational capacity of the various networks used in the simulation experiments, we applied them to the MNIST digit classification task. This is a widely used, basic benchmarking task in computer vision and AI.

For the MB network we employed one epoch, because more epochs did not change the performance. Below, we report on two different settings for the network. The first setting is equal to that in the original experiments in[12], with 5,000 Kenyon cells and 4 active connections (nonzero weights) from the projection neurons to the Kenyon cells. We also show the results for a setting of 20,000 Kenyon cells and 10 active connections. The networks had 10 output neurons. When the network was presented with the digit '5', the top 50 active connections of the corresponding MB output neuron were set to 0. After training, the output neuron with the lowest activity was selected as the predicted class.

The small neural networks used in the proposed strategy were trained with backpropagation for 5 epochs (full passes over the training data). The loss function was the cross entropy function, and the Adam optimizer was used with a learning rate of 10^{-3}. We did not perform any hyperparameter optimization.

The results of the different networks are shown in the following table, with the average and standard deviation of the results over 10 different training runs. It leads to the following observations. First, the performances of the backpropagation-based neural networks are substantially better than those of the MB networks. Second, networks with more parameters obtain a better performance. Specifically, the still small 42.3kB attention network obtains a better performance than the even more compact, 3.4kB network used in most robotic experiments. It even approaches the performance of the much larger "Simple network" that was used in the experiments of the simplified visual environment (SI-5).

Network type	Classification performance
MB network, 5,000 Kenyon cells, 4 active connections from PN to each KC.	47.38% $\pm$ (1.31)
MB network, 20,000 Kenyon cells, 10 active connections from PN to each KC.	52.36% $\pm$ (2.71)
Compact network (5 epochs) – 3.4kB	79.57% $\pm$ (1.95)
Attention network (5 epochs) – 42.3kB	95.97% $\pm$ (0.23)
Simple network (5 epochs) – 6.4MB	**96.67% $\pm$ (0.30)**

Compact network

Layer (type)	Depth idx	Output shape	Param #
Conv2d	1-1	[1, 2, 48, 450]	152
Conv2d	1-2	[1, 2, 12, 113]	102
Conv2d	1-3	[1, 2, 1, 26]	578
Conv2d	1-4	[1, 2, 1, 7]	6
Linear	1-5	[1, 2]	30
**Total**			**868**

Metric	Value
Total params	868
Total mult-adds (M)	3.44
Parameters	3.39kB

Attention network

Layer (type)	Depth idx	Output shape	Param #
InceptionModule2	1-1	[1, 14, 48, 450]	—
└─ Conv2d	2-1	[1, 4, 48, 450]	304
└─ Conv2d	2-2	[1, 4, 96, 900]	112
└─ Conv2d	2-3	[1, 4, 48, 450]	148
└─ Conv2d	2-4	[1, 2, 48, 450]	8
└─ Conv2d	2-5	[1, 4, 48, 450]	112
└─ SpatialAttention	2-6	[1, 1, 48, 450]	—
└─ Conv2d	3-1	[1, 1, 48, 450]	98
└─ Sigmoid	3-2	[1, 1, 48, 450]	—
InceptionModule2	1-2	[1, 14, 12, 113]	—
└─ Conv2d	2-7	[1, 4, 12, 113]	1,404
└─ Conv2d	2-8	[1, 4, 24, 225]	508
└─ Conv2d	2-9	[1, 4, 12, 113]	148
└─ Conv2d	2-10	[1, 2, 12, 113]	30
└─ Conv2d	2-11	[1, 4, 12, 113]	508
└─ SpatialAttention	2-12	[1, 1, 12, 113]	—
└─ Conv2d	3-3	[1, 1, 12, 113]	98
└─ Sigmoid	3-4	[1, 1, 12, 113]	—
Conv2d	1-3	[1, 8, 6, 57]	2,808

Layer (type)	Depth idx	Output shape	Param #
Conv2d	1-4	[1, 4, 1, 52]	1,156
Linear	1-5	[1, 16]	3,344
Linear	1-6	[1, 2]	34
**Total**			**10,820**

Metric	Value
Total params	10,820
Total mult-adds (M)	30.88
Parameters	42.27kB

Simple network

Layer (type)	Depth idx	Output shape	Param #
Conv2d	1-1	[1, 10, 100, 256]	760
Conv2d	1-2	[1, 10, 25, 64]	2,510
Linear	1-3	[1, 100]	1,600,100
ReLU	1-4	[1, 100]	—
Linear	1-5	[1, 2]	202
**Total**			**1,603,572**

Metric	Value
Total params	1,603,572
Total mult-adds (M)	25.07
Parameters	6.12 MiB

SI-13: Comparing compact and attention networks in the same outdoor test dataset

Two networks were proposed for visual homing in the main article: the lightweight compact network and the slightly larger attention network. In Figure 3b,c and Extended Data Table 1, the performance of the attention network was quantitatively compared with the compact network using different learning setups (both onboard and offboard) in the Cyberzoo test arena. In that structured environment, both networks performed well, and ground truth was obtained via the OptiTrack motion capture system.

However, the attention network possesses more parameters and a more advanced network architecture; thus, it is expected to be more robust and powerful than the compact network. To understand how the attention network outperforms the compact network in a challenging environment, we tested both networks on the same learning and homing dataset from the outdoor UVV environment. This dataset, referred to as "GoodDay," was obtained during two of the five full flight experiments originally using the attention network.

Ground truth generation Unlike in the Cyberzoo, where accurate ground truth position and heading can be continuously tracked, we had to rely on manual labelling for this outdoor dataset. We utilized a subset of images gathered during the GoodDay test, specifically selecting only those where the landing/launch pad was visible (see Figure SI-31a) to manually label for obtaining the ground truth. Due to the vertical FoV limitation ($45\ deg$), the ground is typically not visible when the drone is within 2.4 meters of the target (flying at an altitude of 1 m). Conversely, the small pad is not visible when the drone is too far away. This limited the total number of useful images for testing. Ultimately, from the two flights, 26 valid images were labelled and used for evaluation. Furthermore, as only the direction could be reliably labelled using this method, we only compared the direction error.

Training loss We first performed the same self-supervised training process offboard and offline using the learning dataset (as mentioned in Methods). Figure SI-31b shows the loss during the training process. Three losses are shown: the direction and distance losses are calculated for reference purposes, whereas the training loss (calculated by directly computing the mean squared error between the ground truth label and the output) is the actual loss used for backpropagation. All losses for both networks decreased over time, indicating that both networks were able to learn from the image samples gathered during the learning flight. As expected, the attention network demonstrated better learning capabilities, evidenced by a quicker decrease in loss and convergence to a smaller final value.

Testing error Furthermore, we performed predictions on the test dataset using both networks and calculated the absolute angle error using the manually generated ground truth labels. The attention network performed well, with most errors remaining under $50\ deg$, while the compact network performed poorly, as indicated by large angle errors (greater than $90\ deg$), which can lead to navigation failure.

Conclusion In this challenging outdoor environment, the compact network suffers from issues with both learning capacity and generalization. It does not generalize well to data outside the immediate learning set. This can be attributed to its simple structure, small number of parameters, and the limited amount of information available from landmarks (as only a few objects on the ground were available as landmarks, shown in Figure SI-31a). However, its high efficiency and ability to work in all indoor environments and some structured outdoor environments (Figure 4c) can still make it a good choice when deploying in new, relatively structured, and feature-rich environments.

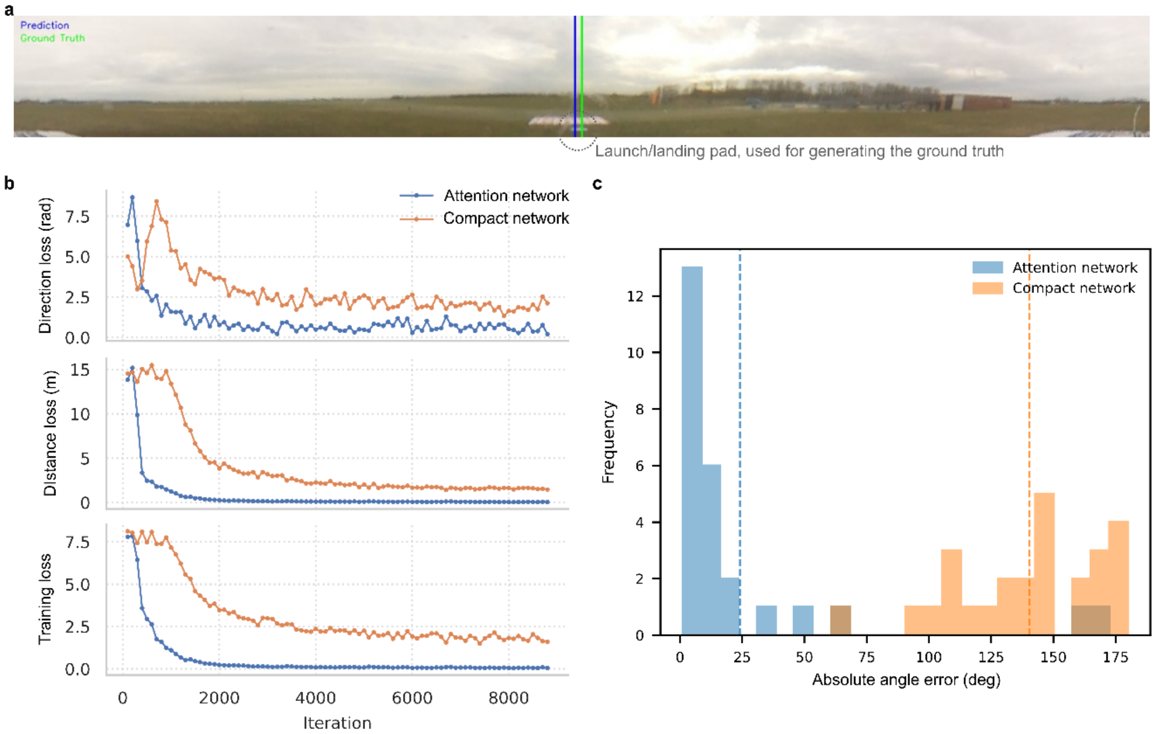

Figure SI-31: Comparison between compact and attention networks in the outdoor GoodDay dataset. a. An example image from the test dataset. The ground truth (green line) is manually labelled using the visual cue of the launch/landing pad (indicated by the grey dashed circle). The blue line shows the angle prediction from the attention network. **b.** Learning loss curves. The direction and distance losses are calculated for reference to monitor specific performance components, whereas the training loss (calculated via mean squared error between the predicted vector and the ground truth) is the actual objective used for backpropagation. While both networks show learning progress (decreasing loss), the attention network (blue) loss drops faster than the compact network (orange) in all categories and converges to a smaller value. **c.** Comparison of the absolute angle error on the test dataset ($n = 26$). The attention network performs well, with most errors remaining under 50 deg, while the compact network performs poorly, as indicated by large angle errors (greater than 90 deg), which lead to navigation failure.

SI-14: Visual homing in different simulated environments

In Bee-Nav, visual homing is performed with small deep neural networks. An important factor for the success of visual homing will be whether such networks will be able to learn accurate home vector prediction in different environments. Given the high representational capacity of deep neural networks, we expect that deep neural networks will be able to extract visual landmark information when present in the images. Of course, this ability will depend on the neural network's architecture and complexity.

In this section, we assess the capabilities of the small 42.3kB attention neural network beyond the experiments in the real world and in the simulated forest. Specifically, we performed additional simulation experiments in environments that were visually distinct from the simulated forest environments reported on in the main article. Figure SI-32 shows the different simulation environments that were used in these additional experiments.

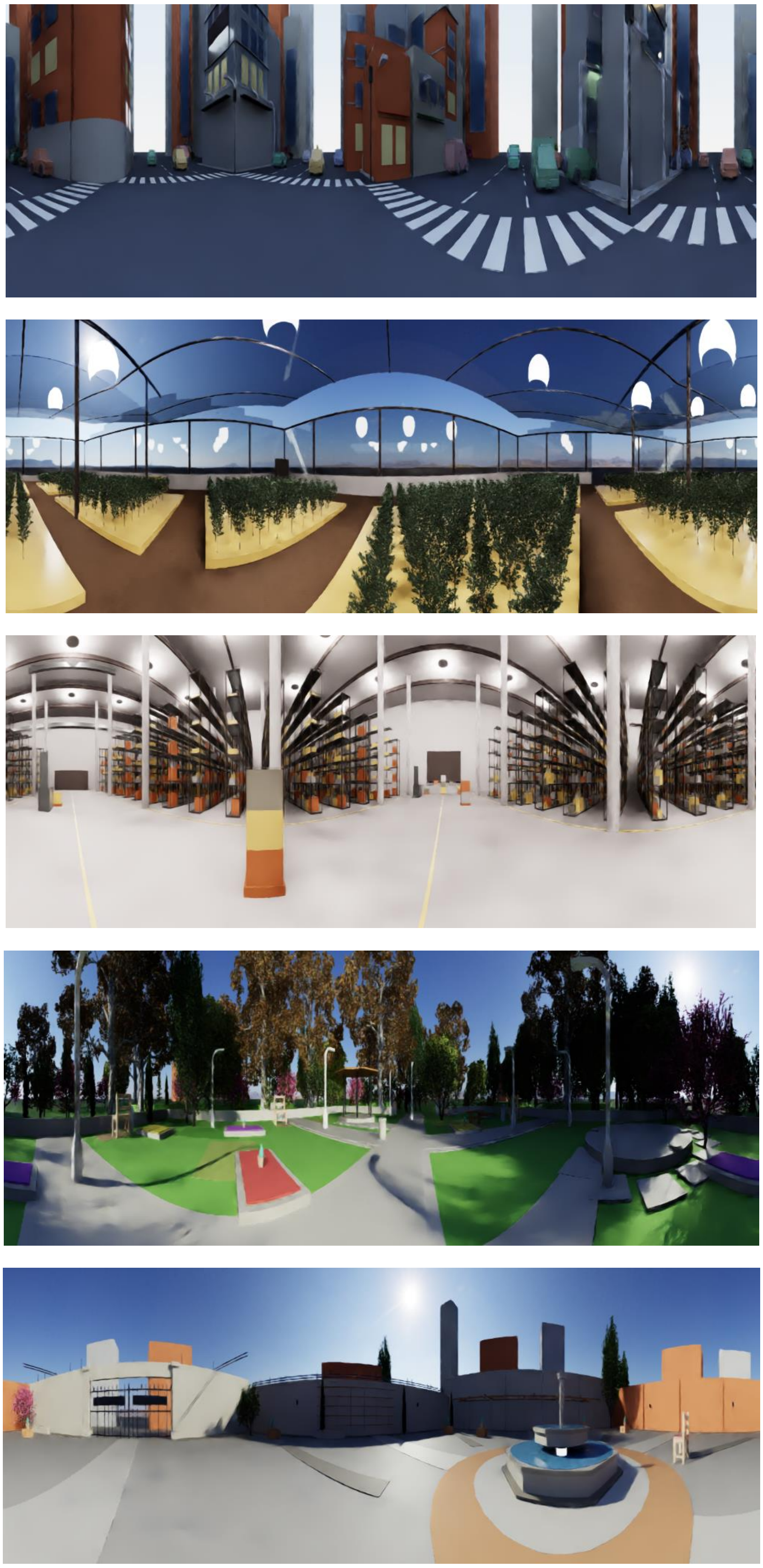

Figure SI-32: Five simulated outdoor and indoor environments that are visually distinct from the simulated forest for additional visual homing experiments.

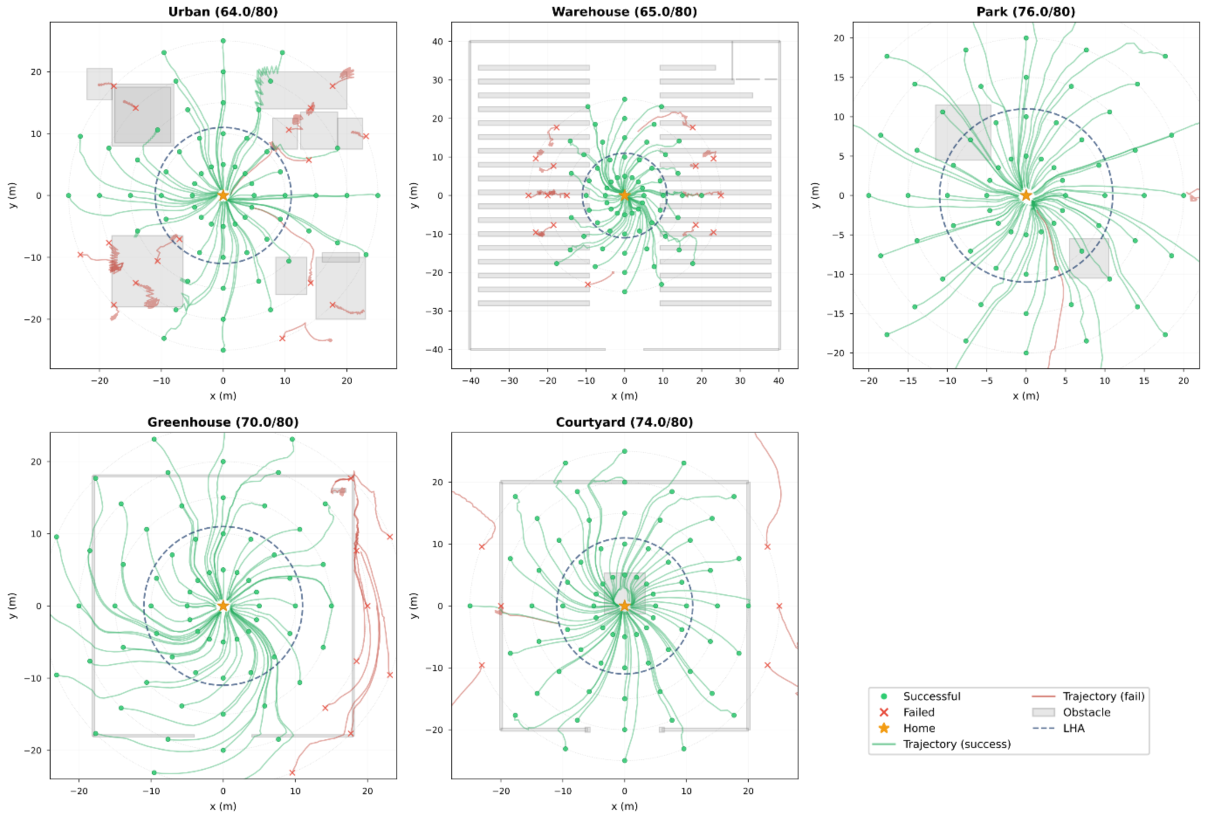

Figure SI-33: Visual homing trajectories in the five simulated environments. Green trajectories represent successes, red trajectories failures. Obstacles are shown in grey.

Figure SI-33 shows the results of the visual homing experiments in these environments, with successful runs in green and failed runs in red. Obstacles are shown in grey. The trajectories show that the neural network can successfully learn to home in these visually very different environments. The failed runs are due to obstacles blocking the view outside of the learned homing area. Please note that no obstacle avoidance was used in these additional experiments, so the camera is also sometimes located inside of obstacles.

Although this is an encouraging result, further robotic experiments will be necessary to reveal the main challenges arising in the real world. For instance, in the simulated urban environment, the cars are not moving, and light conditions are not changing over time. Further data augmentations or extensions to the approach may be necessary to improve robustness to such conditions.

References

1. Degen, J. *et al.* Exploratory behaviour of honeybees during orientation flights. *Anim. Behav.* 102, 45–57 (2015).

2. Benhamou, S. How to reliably estimate the tortuosity of an animal's path:: straightness, sinuosity, or fractal dimension? *J. Theor. Biol.* 229, 209–220 (2004).

3. Mur-Artal, R. & Tardós, J. D. Orb-slam2: An open-source slam system for monocular, stereo, and rgb-d cameras. *IEEE transactions on robotics* 33, 1255–1262 (2017).

4. Sharafutdinov, D. *et al.* Comparison of modern open-source visual SLAM approaches. *J. Intell. Robot. Syst.* 107, 43 (2023).

5. Herrera-Granda, E. P., Torres-Cantero, J. C., Rosales, A. & Peluffo-Ordóñez, D. H. A comparison of monocular visual SLAM and visual odometry methods applied to 3D reconstruction. *Applied Sciences* 13, 8837 (2023).

6. Heinze, S., Narendra, A. & Cheung, A. Principles of insect path integration. *Current Biology* 28, R1043--R1058 (2018).

7. Delmerico, J. & Scaramuzza, D. A benchmark comparison of monocular visual-inertial odometry algorithms for flying robots. in *2018 IEEE international conference on robotics and automation (ICRA)* 2502–2509 (2018).

8. Stankiewicz, J. & Webb, B. Using the neural circuit of the insect central complex for path integration on a micro aerial vehicle. in *Conference on Biomimetic and Biohybrid Systems* 325–337 (2020).

9. Wystrach, A., Mangan, M. & Webb, B. Optimal cue integration in ants. *Proceedings of the Royal Society B: Biological Sciences* 282, 20151484 (2015).

10. Merkle, T. & Wehner, R. Desert ants use foraging distance to adapt the nest search to the uncertainty of the path integrator. *Behavioral Ecology* 21, 349–355 (2010).

11. Wang, Z., Chen, X., Okada, R., Walter, S. & Menzel, R. Encoding and decoding of the information in the honeybee waggle dance. *Behav. Ecol. Sociobiol.* 79, 53 (2025).

12. Gattaux, G. G., Serres, J. R., Ruffier, F. & Wystrach, A. Visual Homing in Outdoor Robots Using Mushroom Body Circuits and Learning Walks. *arXiv preprint arXiv:2507.09725* (2025).

13. Gattaux, G. G., Wystrach, A., Serres, J. R. & Ruffier, F. Route-centric ant-inspired memories enable panoramic route-following in a car-like robot. *Nat. Commun.* 16, 8328 (2025).
